# Biclustering via optimal re-ordering of data matrices in systems biology: rigorous methods and comparative studies

**DOI:** 10.1186/1471-2105-9-458

**Published:** 2008-10-27

**Authors:** Peter A DiMaggio, Scott R McAllister, Christodoulos A Floudas, Xiao-Jiang Feng, Joshua D Rabinowitz, Herschel A Rabitz

**Affiliations:** 1 Department of Chemical Engineering, Princeton University, Princeton, NJ, USA; 2 Department of Chemistry, Princeton University, Princeton, NJ, USA

## Abstract

**Background:**

The analysis of large-scale data sets via clustering techniques is utilized in a number of applications. Biclustering in particular has emerged as an important problem in the analysis of gene expression data since genes may only jointly respond over a subset of conditions. Biclustering algorithms also have important applications in sample classification where, for instance, tissue samples can be classified as cancerous or normal. Many of the methods for biclustering, and clustering algorithms in general, utilize simplified models or heuristic strategies for identifying the "best" grouping of elements according to some metric and cluster definition and thus result in suboptimal clusters.

**Results:**

In this article, we present a rigorous approach to biclustering, OREO, which is based on the Optimal RE-Ordering of the rows and columns of a data matrix so as to globally minimize the dissimilarity metric. The physical permutations of the rows and columns of the data matrix can be modeled as either a network flow problem or a traveling salesman problem. Cluster boundaries in one dimension are used to partition and re-order the other dimensions of the corresponding submatrices to generate biclusters. The performance of OREO is tested on (a) metabolite concentration data, (b) an image reconstruction matrix, (c) synthetic data with implanted biclusters, and gene expression data for (d) colon cancer data, (e) breast cancer data, as well as (f) yeast segregant data to validate the ability of the proposed method and compare it to existing biclustering and clustering methods.

**Conclusion:**

We demonstrate that this rigorous global optimization method for biclustering produces clusters with more insightful groupings of similar entities, such as genes or metabolites sharing common functions, than other clustering and biclustering algorithms and can reconstruct underlying fundamental patterns in the data for several distinct sets of data matrices arising in important biological applications.

## Background

Problems of data organization and data clustering are prevalent across a number of different disciplines. These areas include pattern recognition [1], image processing [2], information retrieval [3], microarray gene expression [4], and protein structure prediction [5,6], just to name a few. The goal of data clustering, regardless of the application, is to organize data in such a way that objects which exhibit "similar" attributes are grouped together. The definition of similarity depends on the application and may correspond to the direct comparison of values or the degree of correlation among trends or patterns of values.

Several methods have been proposed for the clustering of large-scale, dense data. The most common approaches to the data clustering problem are typically categorized as hierarchical [4] or partitioning [7] clustering. Although algorithms to identify the optimal solutions to these categories of problems do exist [8-10], they are frequently solved using heuristic search techniques that result in suboptimal clusters because the comparisons between terms are evaluated locally. Various other frameworks for data clustering have been proposed, including model-based clustering [11,12], neural networks [13], simulated annealing [14], genetic algorithms [15,16], information-based clustering [17], decomposition based approaches [18-20], and data classification [21,22]. The field of rearrangement clustering has emerged as an effective technique for *optimally *minimizing the sum of the pairwise distances between rearranged rows and columns. The bond energy algorithm (BEA) was originally proposed as a method for finding "good" solutions to this problem [23] and it was subsequently discovered that this problem could be formulated as a traveling salesman problem (TSP) which can be solved to optimality [24,25] using existing methods.

If a gene is involved in more than one biological process or belongs to a group of genes that are coexpressed under limited conditions, then alternative cluster definitions and clustering techniques are required [26]. A bicluster is defined as a submatrix which spans a certain set of genes (rows) and certain set of conditions (columns). Common elements can be shared among biclusters and there is no requirement that all members of the original matrix are classified in a bicluster. Several different models and algorithms have been developed for this NP-hard problem [27]. To generate biclusters within a reasonable amount of time, many existing techniques either employ heuristic methods for generating good solutions or simplify the problem representation, such as discretizing the expression level.

The Cheng and Church [27] and cMonkey [28] biclustering algorithms are iterative processes and allow for integration of other data types since they do not transform the data. The Cheng and Church algorithm uses a greedy heuristic to solve an optimization problem based on the mean square residue, which provides a measure of deviation from the actual value of an element and its expected value based on the row, column, and bicluster mean [27]. Other methods for biclustering, such as plaid [26] and spectra models [29], are related to projection methods which regenerate the data matrix by biclusters. The plaid model expresses the value of each element in the gene expression data as a series of additive layers [26] and the spectra model uses singular value decomposition to identify eigenvectors that reveal the existence of checkerboard structures within the rearranged genes and conditions [29]. Another matrix factorization based method, nsNMF [30], utilizes non negative matrix factorization with non-smoothness constraints to identify block-structures (biclusters) in gene expression data for a given factorization rank. In contrast to the plaid model, which focuses on the uniformity of expression levels, biclusters defined by order-preserving submatrices focus on the relative order of the columns [31] in an attempt to identify biclusters with coherent evolutions. The biclustering methods Bimax [32] and Samba [33] discretize the expression level which allows them to enumerate a large number of biclusters in less time than more complicated models. To complement the assortment of problem representations for biclustering, there have been a variety of algorithmic approaches developed to solve these models of varying complexity, such as zero-suppressed binary decision diagrams [34], evolutionary algorithms [35,36], Markov chain Monte Carlo [28], bipartite graphs [33], and 0–1 fractional programming [37]. An excellent review of different bicluster definitions and biclustering algorithms can be found in [38].

In this article, we introduce a biclustering algorithm which iteratively utilizes optimal re-ordering to cluster the rows and columns of dense data matrices in systems biology. We present several objective functions to guide the rearrangement of the data and two different mathematical models (network flow and traveling salesman problem) to perform the row and column permutations of the original data matrix. We demonstrate that this global optimization method provides a closer grouping of interrelated entities than other clustering and biclustering algorithms, produces clusters with insightful molecular functions, and can reconstruct underlying fundamental patterns in the data for several distinct sets of data matrices arising in important biological applications.

## Results and discussion

In this section, we present the results for our proposed biclustering method for a variety of interesting systems. We first demonstrate the effectiveness of the proposed algorithm by analyzing systems that can be manually or visually assessed. For this purpose, we chose to examine (a) a small data matrix consisting of metabolite concentration data and (b) an image reconstruction problem, which allows for visual inspection of the results. We then apply the proposed methodology to larger systems corresponding to (c) synthetic data with implanted biclusters and gene expression data for (d) colon cancer data, (e) breast cancer data, as well as (f) yeast segregant data. For each of these data sets, we draw comparisons with several other clustering and biclustering techniques.

### Case Study 1: Metabolite Concentration Data

**Figure 1:**
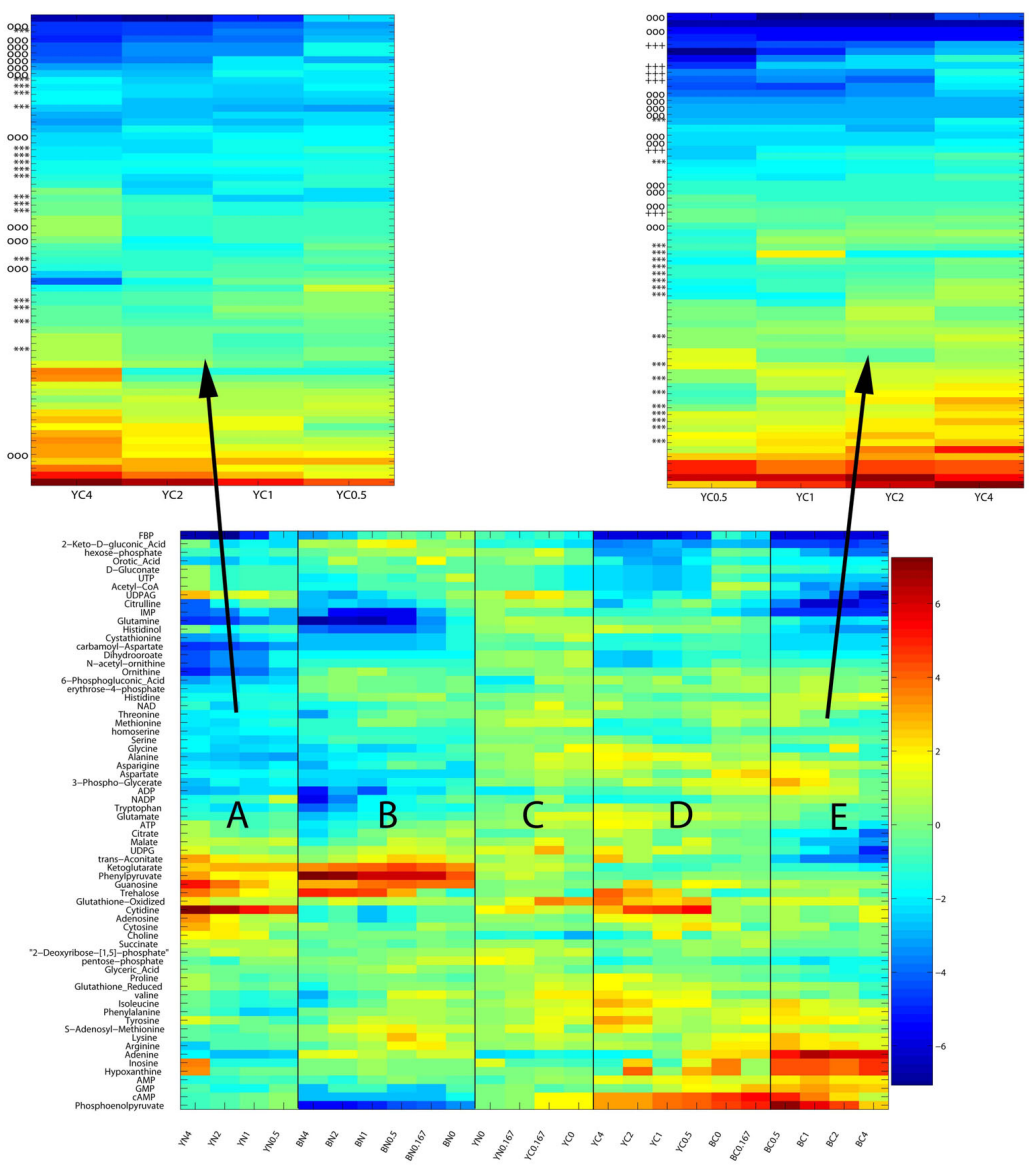
**Re-ordered metabolite concentration data**. In the lower matrix, the partitioning of columns into regions A, B, C, D, and E after computing the cluster boundaries is presented given the optimal re-ordering over all conditions. The upper matrices illustrate the subsequent optimal ordering of the metabolites over the regions A and E. The labels on the x-axis denote starvation conditions, where "YN" and "YC" denote the starvation of *S. cerevisiae*, Y, of nitrogen (N) and carbon (C), respectively. Similarly, the x-axis labels "BN" and "BC" denote the starvation of *E. coli*, B, of nitrogen (N) and carbon (C), respectively. The numbers succeeding these x-axis labels denote the time, in hours, at which the concentration was measured. The y-axis labels in the bottom matrix provide the names of the metabolites. In the upper two matrices, the relative groupings of related metabolites are illustrated using the labels "***" for amino acid metabolites, "ooo" for biosynthetic intermediates, and "+++" for TCA compounds. The values in the data matrix correspond to the logarithm (base 2) of the fold-change in relative metabolite concentration as measured using liquid chromatography and tandem mass spectrometry. Fold-change is relative to exponentially growing cells. Blue colors indicate fold decreases and red colors indicate fold increases.

The proposed method was tested on data comprised of concentration profiles for 68 metabolites (the rows of the data matrix) recorded over time (columns of the data matrix) for the organisms *E. coli *and *S. cerevisiae *under the conditions of nitrogen and carbon starvation for both organisms [39]. The concentration changes were dynamically measured using liquid chromatography-tandem mass spectrometry. We applied our biclustering algorithm to this data using the objective function defined in Eq. 3. The re-ordering problem for the columns was solved to global optimality using the mixed-integer linear programming algorithm in CPLEX [40] in 2.7 seconds on an Intel 3.0 GHz Pentium 4 processor. The optimal ordering for the columns using the objective function in Eq. 3 is shown in Figure 1, where the top four cluster partitions for the columns are denoted by the solid vertical lines.

It is interesting to note that the two most significant cluster boundaries perfectly partition subsets of the *E. coli *and *S. cerevisiae *conditions. An interesting feature of the column rearragnement is that all the nitrogen starvation conditions occupy one half of the matrix and the carbon starvation conditions occupy the remaining half of the matrix. The regions between these cluster boundaries, labeled A, B, C, D, and E in Figure 1, are also optimally re-ordered using the proposed method. For the sake of brevity, let us consider the results obtained from optimally re-ordering the submatrices for region E, as shown in the enlarged regions of Figure 1. The submatrix for region E was optimally re-ordered in 0.18 CPU seconds. The optimally re-ordered metabolites for region E over the conditions of carbon starvation in *E. coli *yields an excellent grouping of amino acid and TCA metabolites. In a cluster of 27 metabolites, 16 are amino acids (out of a total of 19 amino acids in the data) and 8 are ordered consecutively: serine, glycine, valine, glutamate, tryptophan, alanine, threonine, and methionine (see the "***" symbols in Figure 1). This richness of amino acid metabolites is consistent with the observation that amino acids tend to accumulate during carbon starvation [39]. Another interesting feature is that four out of the six TCA metabolites (trans-aconitate, citrate, malate, and acetly-coa, represented by the "+++" symbols in Figure 1) are within six positions of each other in the optimal ordering. The biosynthetic intermediates also order well for this submatrix (as shown by the "ooo" symbols in Figure 1), where all twelve are placed in the top half of the re-ordered matrix, which is rich in metabolites that are decreasing in concentration. An interesting observation is the final position of FBP relative to phospoenolpyruvate (PEP), which are exactly opposite each other in the re-ordered matrix. PEP is a positive regulator of pyruvate kinase, which is the major enzyme consuming PEP [39]. Since carbon-starvation resulted in a decrease of FBP, this presumably down-regulates the activity of pyruvate kinase, which in turn results in PEP accumulation.

We compared our findings with the results for hierarchical clustering [4] applied to the metabolite concentration data [39]. The hierarchical clustering placed the majority of amino acids in the top half of the arranged matrix, with the largest consecutive ordering of amino acids being alanine, glutamate, threonine, methionine, and serine, which is a less significant clustering than those found in region E for OREO. The TCA cycle compounds were also not found to be grouped as well for hierarchical clustering as they were for OREO, where four TCA cycle compounds (aconitate, malate, citrate, and succinate) were assigned to a cluster of ten metabolites [39]. We also optimally re-ordered the hierarchical clustering leaves using the TreeArrange algorithm [41] to see if the clustering of related metabolites would improve. The most notable improvement in the results of the optimal leaf ordering are a grouping of 6 amino acid metabolites out of 9 metabolites (threonine, glutamate, tryptophan, asparigine, alanine, glycine) and an ordering where 8 biosynthetic intermediate metabolites were found in a span of 9 metabolites. Overall, when compared to hierarchical clustering, with and without optimal leaf ordering, OREO arranges the metabolites in an order which more closely reflects their known metabolic functions.

The objective function values for Eq. 3 were evaluated for the final ordering as provided by the hierarchical clustering results and then compared to the optimal values that were determined using our method over all columns and rows (shown in Table 1). The "Gap" column in Table 1 is a standard measure for quantifying the deviation of an ordering from optimality. Based on Table 1, the final ordering provided by the hierarchical results, with and without optimal leaf ordering, are suboptimal with respect to the squared difference objective function.

**Table 1:** Comparison between optimal objective and hierarchical objective value.

Data Set	Dimension	Optimal Objf	HC Objf	HC (Opt. Order) Objf
Metabolite	Rows	2,662.8	3,783.2 (29.6%)	3,550.2 (25.0%)
Concentration [39]	Columns	1,753.0	2,044.3 (14.2%)	1,865.8 (6.0%)

Colon	Rows	26,602.6	40,878.5 (34.9%)	35,637.0 (25.4%)
Cancer [44]	Columns	32,174.0	43,627.2 (26.3%)	39,138.3 (17.8%)

Breast	Rows	27,613.8	38,572.5 (28.4%)	36,182.4 (23.7%)
Cancer [48]	Columns	42,711.4	49,064.7 (12.9%)	48,553.1 (12.0%)

Yeast	Rows	82,162.4	120,429.0 (31.8%)	111,612.1 (26.4%)
Segregant [50]	Columns	124,441.0	154,353.3 (19.4%)	154,353.3(19.4%)

"Objf" denotes the objective function for the squared difference metric (see Eq. 3) and hierarchical clustering (using the Euclidean metric), with and without optimal leaf ordering. "HC" corresponds to hierarchical clustering [4] and "HC (Opt. Order)" corresponds to the optimal leaf ordering of hierarchical clustering using TreeArrange [41].

### Case Study 1: Results and Comparisons with Other Biclustering Algorithms

Since the rearranged data appears to naturally form biclusters, we compared the results for OREO with the biclustering algorithms ISA, Cheng and Church's, OPSM, BiMax, SAMBA, and nsNMF on the metabolite concentration data set. Each algorithm was run using the default parameter values, which were adjusted in the event that no biclusters were found. The biclustering results were visualized using the BiVoc algorithm [42] and are provided along with a complete description of the results obtained for each method [see Figures S.1 through S.7 in Additional file 1].

The best results were reported by Cheng and Church's Algorithm [27] and nsNMF. For Cheng and Church's algorithm, the best bicluster consisted of 30 metabolites, of which 15 were assigned to the amino acid category [39] over various conditions related to carbon and nitrogen starvation in *E. coli *and *S. cerevisiae*. The longest consecutive ordering of amino acids within this bicluster are serine, methionine, threonine, glutamate, and alanine, which is exactly the same as that reported in the hierarchical clustering results. The majority of the other metabolites in this bicluster are biosynthetic intermediates. The biclustering algorithm nsNMF was applied to the metabolite concentration data set for 100 runs using a cophenetic correlation coefficient of *k *= 2 [30]. Sorting the starvation conditions using the first and second basis metabolites also perfectly separates the nitrogen and carbon starved samples for both basis metabolites, which is consistent with the findings of OREO. When sorting the metabolites using the first basis condition, there is an excellent grouping of 15 amino acid metabolites within a span of 22 metabolites. The metabolites sorted by the second basis condition did not yield any significant grouping of related metabolites.

### Case Study 2: Image Reconstruction (Lenna Matrix)

**Figure 2:**
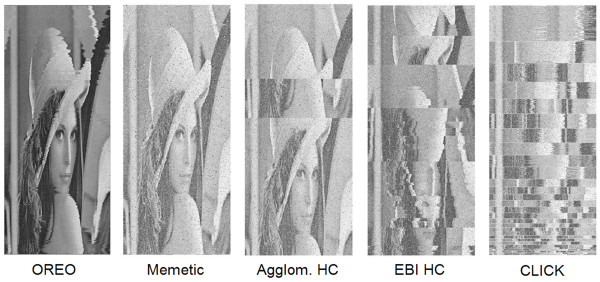
**Re-ordering of Whole Lenna Image with Noise**. The clustering results for OREO, a Memetic algorithm, EBI hierarchical clustering, agglomerative hierarchical clustering, and CLICK for the replicated Lenna image after introducing noise into the pixels (see text for details). The original image is again recovered by OREO and the Memetic algorithm, whereas the other methods cannot reproduce the correct ordering.

We also applied the proposed method to a data matrix representing an image, commonly referred to as the "Lenna image", which has been extensively studied in the image processing community. Although the pixels of an image are very different than patterns observed in systems biology data, studying such a matrix allows us to visually assess the ability of the proposed approach. In the experiment presented in [43], the original image, which consists of 512 by 512 pixels, was elongated by replicating it 10 times to create a 5,120 by 512 matrix. The optimal ordering of this replicated image should result in a stretched version of the original image. The results for a Memetic algorithm, CLICK, and two other methods based on hierarchical clustering were presented in this study [43] and here we compare our results to these findings. OREO was able to recover the correct ordering for the original image and a subset of the original image [see Figures S.8 and S.9 in Additional file 1]. This image was also examined after introducing two types of noise: (1) modifying *every *pixel by a random value less than 10% of the maximum pixel intensity (255) and (2) assigning a random value between 0 and 255 to 10% of the pixels (e.g., 262,144 of the pixels). The optimal ordering determined by OREO is presented in Figure 2, where we see that we again recover the correct image. The Memetic algorithm is able to recover the original image and the agglomerative clustering algorithm performs slightly worse given this noise level, as the misplaced subsection of the image has become larger (compare Figures 2 and S.8). As in the case without any noise (see Figure S.8), the EBI hierarchical clustering algorithm and CLICK chop the original image into many disjunct subsections. Although this example does not correspond to biological data, it illustrates the applicability of OREO for other systems.

### Case Study 3: Synthetic Data with Implanted Biclusters

We also tested our proposed methodology on a data set corresponding to synthetic gene expression data created by an artificial model [32]. In these data sets, both constant and additive biclusters of varying degree of overlap were implanted into a simulated matrix and subjected to different levels of noise. The biclustering methods BiMax, ISA, SAMBA, Cheng and Church's, OPSM, xMotif, and hierarchical clustering were applied to these data matrices and the results were assessed based on two metrics: (1) the average bicluster relevance and (2) the average module recovery, as defined in [32]. The average bicluster relevance is a quality measure for the biclusters that are produced by a particular method and the average module recovery is a measure of how well a particular method is at finding all of the implanted biclusters. We applied OREO to the four sets of synthetic data (provided at ): (1) non-overlapping constant biclusters, (2) non-overlapping additive biclusters, (3) overlapping constant biclusters, and (4) overlapping additive biclusters, all subjected to varying levels of noise estimated from a normal distribution. The average bicluster relevance and average module recovery were computed for OREO for each of these data sets [see Figures S.10 through S.17 in Additional file 1].

When analyzing the non-overlapping and constant bicluster data sets, the biclusters produced by OREO have a perfect score for average bicluster relevance and average module recovery, as shown in Figures S.10 and S.11. As can be seen in Figure 2(a) in [32], only hierarchical clustering performs as well as OREO for this data set. The biclusters produced by ISA have a perfect score for average bicluster relevance but have slightly worse scores for the average module recovery. BiMax also scores well for these two metrics, but it is observed in Figure 2(a) in [32] that its performance decreases with increasing noise level. The biclusters produced by the other methods do not score very well for this set of synthetic data.

For the non-overlapping and additive bicluster data sets, OREO consistently produces biclusters that have an average bicluster relevance score greater than 0.90 (see Figure S.12). In Figure 2(b) from [32], it is seen that ISA and SAMBA produce biclusters that have almost perfect average bicluster relevance scores. The average bicluster relevance for hierarchical clustering is shown to consistently decrease with increasing noise level for these additive biclusters. In terms of average module recovery, it is seen in Figure S.13 that OREO again consistently scores above 0.92 over the varying levels of noise. The average module recovery of BiMax and ISA are shown to be comparable to that of OREO as observed in Figure 2(b) in [32], whereas the other methods do not score as well for the additive biclusters subject to varying degrees of noise. For the data sets corresponding to overlapping constant biclusters, the average bicluster recovery and average module recovery follow similar trends (see Figures S.14 and S.15); the scores initially start at one (i.e., non-overlapping), slightly fall for overlapping degrees of 1 and 2 elements, then rise back to a score of one for the overlapping degrees ranging from 3 to 7 and then finally descend at an overlap degree of 8 elements. From Figure 2(c) in [32], it is shown that BiMax produces biclusters with perfect scores for the average bicluster relevance and average module recovery, SAMBA produces biclusters with perfect scores for average bicluster relevance but significantly poorer scores for average module recovery (all scores with the exception of the non-overlapping instance are below 0.8), and ISA performs slightly better than OREO in terms of average bicluster relevance for the data matrices with overlapping degrees of 1 and 2 elements. It is observed that the remaining methods produce clusters that consistently score less than 0.7.

When examining the overlapping and additive bicluster data sets, the average bicluster relevance for OREO is slightly higher than 0.8 on average and the average module recovery is about 0.9 on average, as shown in Figures S.16 and S.17. From Figure 2(d) in [32], it is seen that BiMax produces biclusters that have perfect scores for the average bicluster relevance and average module recovery. SAMBA also performs well for the additive biclusters, with the exception of very low scores (less than 0.6) for an overlap degree of 7 elements. ISA performs comparably to OREO for the average bicluster recovery but is shown to decrease in performance with respect to increasing noise level. The results for hierarchical clustering for the overlapping data sets are shown to be much worse than for the non-overlapping data sets and the remaining biclustering methods consistently yield scores less than 0.6 in both metrics.

### Case Study 4: Colon Cancer Data

**Figure 3:**
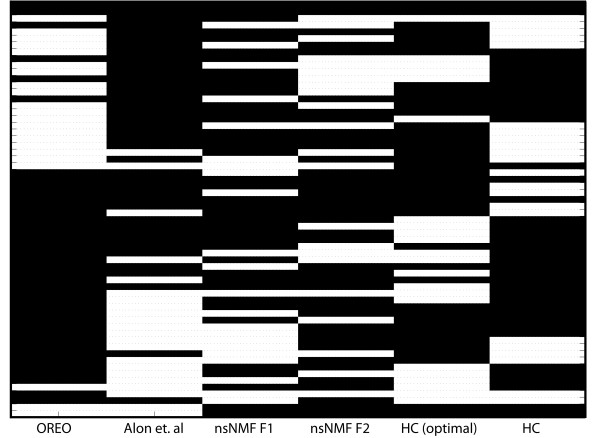
**Partitioning of tumor and normal tissues for colon cancer data**. Illustration of the tumor and normal tissues after re-ordering the tissues for OREO, Alon et al., nsNMF after sorting of the first and second basis genes (as denoted by nsNMF F1 and nsNMF F2, respectively), hierarchical clustering after optimally re-ordering the leaves (HC (opt)), and hierarchical clustering (HC). In the data matrix, the white elements denote normal tissues and the black elements denote tumor tissues. Only OREO and Alon et al. were successful in separating the normal and tumor tissues into two dense regions.

We also tested the proposed method on a standard biclustering sample classification example [38] comprised of gene expression data for 62 colon tissue samples, 22 of which were normal and 40 of which were tumor tissues [44]. In the original work by Alon et al. [44], the 2000 genes with the highest minimal intensity across the samples were examined using a deterministic-annealing algorithm [45]. Two-way clustering was performed on both the genes and the tissue samples and it was found that the algorithm was able to approximately separate the tissues into a normal-rich cluster and a tumor-rich cluster. Figure 3 illustrates the separation of the tissues into tumor-rich and normal-rich regions, where the tumor tissues are in black and the normal tissues in white. Only three normal tissues (N8, N12, N34) were assigned in the tumor-rich tissue region and a total of five tumor tissues (T30, T36, T33, T37, T2) were placed in the normal-rich tissue region [see Table S.1 in Additional file 1]. The clustering of the genes revealed a strong correlation among the ribosomal proteins, where a cluster consisting of 22 ribosomal proteins was discovered [44]. We applied our biclustering method to the same set of 2000 genes of highest minimal intensity and 62 tissue samples using the traveling salesman representation and the objective function defined in Eq. 3. The original data was normalized by performing Z-normalization over all genes and all tissues. The optimal re-ordering for the tissues (or columns) was achieved in a CPU time of 0.17 seconds. The normal and tumor tissue samples were partitioned into normal-rich and tumor-rich regions based on the largest two cluster boundaries. Figure 3 illustrates the partitioning of the tumor and normal tissues and the tissue names in the normal- and tumor-rich regions are provided [see Table S.1 in Additional file 1]. Note that in Figure 3, OREO provides the richest grouping of tumor tissues in comparison to all the other methods. Overall, these results are consistent with the findings of Alon et al. in that N8 and N34 were incorrectly grouped with the tumor tissues and T30, T36, T33, T37, T2 were incorrectly grouped with the normal tissues [see Table S.1 Additional file 1]. The genes (or rows) of the data matrix were then optimally re-ordered over both the corresponding tumor and normal-rich submatrices. To compare our clustering with the results presented in Alon et al., we examined the final orderings of the genes related to ribosomal proteins and growth factors. For the tumor-rich submatrix, OREO organized 30 out of the 48 ESTs homologous to ribosomal proteins into one dense cluster, which is very similar to the findings of Alon et al. [44]. Interdispersed among the ribosomal protein cluster are 6 ESTs homologous to genes that are related to cell growth, such as elongation factors, which is also consistent with previous findings [44,46].

The colon cancer data was also examined using hierarchical clustering [4]. The genes related to ribosomal proteins were clustered together, but only 19 out of the 48 were grouped into a larger cluster with 5 ESTs homologous to genes related to cell growth interdispersed throughout. Figure 3 shows that the separation of normal and tumor tissues was not as consistent for hierarchical clustering, where there are several alternating regions of tumor- and normal-rich tissues. Even after optimally re-ordering the hierarchical clustering leaves using TreeArrange [41], the tumor and normal tissues do not separate into two distinct groupings as shown in Figure 3. The clustering of the 25 ribosomal proteins and cell growth factors do not change after optimal re-ordering of the leaves. In Table 1, we present the deviation from optimality for the ordering reported from hierarchical clustering with reference to the optimal ordering over all columns and rows as determined by our method.

### Case Study 4: Results and Comparisons with Other Biclustering Algorithms

The biclustering algorithm nsNMF [30] was applied to this data set and the separation of the tissues after sorting on the first and second basis genes are presented in Figure 3, where it is shown that both factors fail to separate the normal and tumor tissues into two distinct regions. However, when sorting the genes on the first basis tissue, a cluster of 23 ribosomal proteins and 7 ESTs homologous to genes related to cell growth is discovered with a relative grouping similar to that of OREO. We also examined this data set with the biclustering algorithms ISA, SAMBA, xMotif, OPSM, and Cheng and Church [see Additional file 2] using the default parameters of each method. For all of the biclustering methods, we examined the molecular function and biological process enrichment of the corresponding biclusters using the ontology tool Onto-Express [47], applying a hypergeometric distribution and referencing the calculations by the 2000 genes analyzed. If the algorithm produced more than 15 biclusters, we selected the highest scoring 15 or the first 15 that were reported if no scores were provided. The ontology results for each of the biclustering methods are available [see Additional file 3]. From the ontology analysis, it was found that OREO uncovers several biclusters that are significantly annotated to the molecular function "structural constituent of ribosome", which corresponds to the aforementioned ribosomal proteins that were the focus of discussion in the Alon et al. study. The only other methods that provided biclusters significantly annotated to the molecular function of "structural constituent of ribosome" were nsNMF (after sorting on the first factor) and SAMBA.

### Case Study 5: Breast Cancer Data

The proposed biclustering method was also applied to breast cancer data studied by Van't Veer et al. [48]. In this data matrix, the expression level for approximately 25,000 genes over 98 breast cancer tumors were measured. A supervised clustering method was used to determine the optimal number of reporter genes for classification based on prognosis, ER status, and BRCA1 germline mutation carriers [48]. In this study, it was discovered that about 5,000 of the most significantly regulated genes across the 98 tumor samples, which had at least a two-fold difference and a p-value of less than 0.01 in five or more samples, were effective in separating ER positive from ER negative tumor samples. Missing data values for this matrix were estimated using the k-nearest neighbors approach [49].

**Figure 4:**
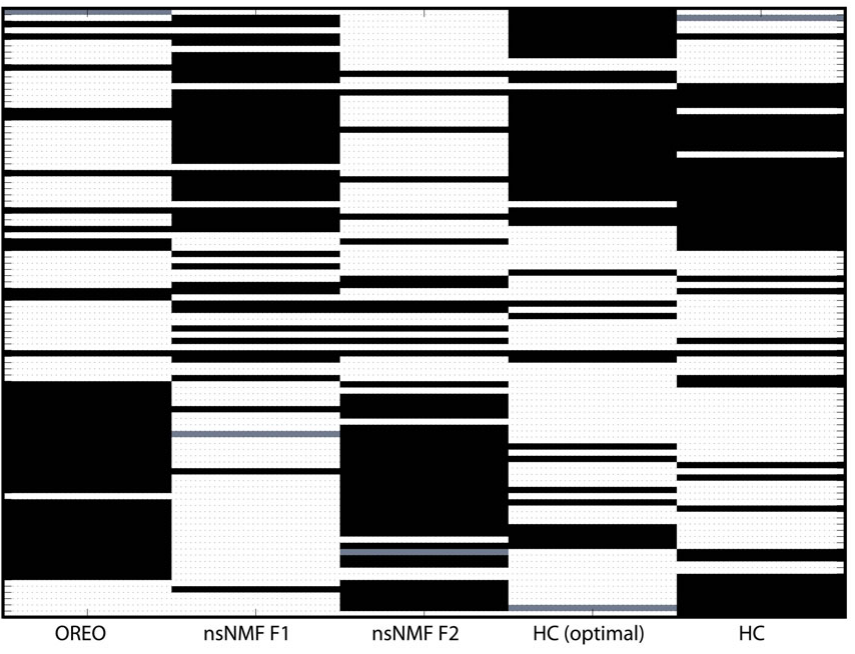
**Partitioning of ER expression tumors for breast cancer data**. Tumor groupings after re-ordering for OREO, nsNMF after sorting of the first and second basis genes (as denoted by nsNMF F1 and nsNMF F2, respectively), hierarchical clustering after optimally re-ordering the leaves (HC (opt)), and hierarchical clustering (HC). In the data matrix, the white elements denote ER positive tumors, the black elements denote ER negative tumors, and the grey element denotes as tumor for which ER expression is unknown. All methods result in a moderately successful separation of ER positive and ER negative tumors, with OREO resulting in the richest grouping of ER negative tumors.

OREO was applied to this set of about 5,000 significant genes and 98 tumor samples and the tumors were re-ordered in 0.09 CPU seconds. In Figure 4 it is shown that the column re-ordering for OREO is fairly successful in partitioning the ER positive and ER negative tumors, with 13 ER negative tumors assigned to the ER positive region and 1 ER positive tumor assigned in the ER negative region. Hierarchical clustering [4] was also applied to the same data matrix and the resulting arrangement of the tumors is shown in Figure 4, where it is shown that there is a reasonable grouping of the ER positive and ER negative tumors, but an overall separation is not achieved. However, the partitioning is enhanced after optimally re-ordering the leaves using TreeArrange, as shown in Figure 4, where 12 ER negative tumors are assigned to the ER positive region and 4 ER positive tumors are assigned in the ER negative region. We examined the clustering of the 550 optimal ER status genes as determined by Van't Veer et al. [48]. On average, OREO required 3115 CPU seconds to optimally re-order these roughly 5,000 genes over the resulting biclusters. We then examined the densest clustering of at least 50 of these genes for OREO and hierarchical clustering, with and without optimal leaf ordering. In other words, we searched for the smallest size neighborhood of genes in which 50 ER status genes were found. For OREO, 50 of the optimal ER status genes were found in a span of only 171 genes (29.2%). For hierarchical clustering, the densest clustering of optimal ER status genes was found to be 53 within a span of 268 genes (19.8%). However, after optimally re-ordering the leaves, a grouping of 56 ER status genes was found in a span of only 172 genes (32.6%). Table 1 illustrates the deviations from optimality for the re-orderings provided by hierarchical clustering, with and without optimal leaf ordering.

### Case Study 5: Results and Comparisons with Other Biclustering Algorithms

The biclustering algorithm nsNMF [30] was also applied to this data set for 100 runs with a cophenetic correlation coefficient of *k *= 2. The ordering of the tumors after sorting on the first and second basis genes are presented in Figure 4, where one can see that the nsNMF algorithm is also fairly successful in partitioning the tumors which exhibit ER expression. Although the orderings are different for sorting on the two factors, both result in 14 ER negative tumors assigned to the ER positive region and 6 ER positive tumors assigned in the ER negative region. The biclustering methods ISA, SAMBA, xMotif, OPSM, and Cheng and Church were applied to this breast cancer data set for comparison using the default parameters [see Additional file 3]. We examined the molecular function and biological enrichment of the corresponding biclusters using Onto-Express [47], using a hypergeometric distribution and referencing the calculations by the 5000 genes analyzed [see Additional file 4]. If the algorithm produced more than 15 biclusters for this data set, we examined the biological enrichment for the highest scoring 15 or the first 15 that were reported if no scores were provided. It is observed from the ontology results [see Table S.3 in Additional file 1] that OREO had uncovered biclusters with a significant enrichment for the molecular functions "MHC class II receptor activity" and "MHC class I receptor activity". It is well-known that MHC proteins are cell-surface glycoproteins that bind peptides within the cell, then bring the peptide to the surface for interaction with T cells, which is part of the mechanism in which the body identifies and responds to foreign antigens. These findings are complemented by several OREO biclusters that are enriched in the biological process "immune response". The only other method which found biclusters annotated to both the molecular process "MHC class I receptor activity" and the biological process "immune response" was OPSM. The algorithms nsNMF and SAMBA also identified biclusters annotated to the "immune response" biological process.

### Evaluation of Biclustering Results in Case Studies 1, 4, and 5 Using Standard Metrics

To assess the quality of the biclusters produced as a function of the input data, we computed the average correlation among the rows and columns as a function of (1) the bicluster area (the number of rows times the number of columns in a bicluster), (2) the number of rows per bicluster, and (3) the number of columns per bicluster for the (a) metabolite concentration data, (b) colon cancer data, and (c) breast cancer data sets. The average correlation values for each of these scenarios were computed [see Figures S.18 through S.29 in Additional file 1].

For the average correlation over the bicluster rows as a function of bicluster area (see Figures S.18, S.22, and S.26), it is observed that OPSM consistently produces biclusters with the highest average row correlation values for the smaller bicluster areas. Note that in each of these data sets, the average correlation values for OPSM are monotonically decreasing as a function of the bicluster area. It is also observed in each of the data sets that OREO produces biclusters that are the largest in area and have average row correlation values ranging between 0.4 and 0.7. In the majority of instances, no other biclustering methods produce biclusters comparable to this size. The average row correlation values for the biclusters produced by ISA range from 0 to 0.7, with an average row correlation of about 0.25 over the data sets. The biclusters produced by SAMBA generally have better average row correlation values than ISA for a given bicluster area. In particular, SAMBA performs well for the breast cancer data set (see Figure S.26) and produces many biclusters with row correlation values that range from 0.18 to 0.84. For the colon cancer data set, SAMBA produces sizable biclusters that are similar in area to those produced by OREO, but the row correlation values for OREO are consistently higher. Cheng and Church's algorithm produces biclusters of consistently lower average row correlation values than the other biclustering methods, with the exception of a few biclusters corresponding to the metabolite concentration data set (in Figure S.18) that have correlation values greater than 0.5. The aforementioned trends are consisently observed for the average correlation over rows as a function of number of rows for all biclustering methods (presented in Figures S.19, S.23, and S.27).

We also examined the average correlation among the bicluster columns as a function of bicluster area, and the results are presented in Figures S.20, S.24, and S.28. In contrast to the row correlation as a function of bicluster area, there were no discernable trends for the correlation of columns as a function of bicluster area for OPSM. In fact, the average correlation values over the columns are not dominant for OPSM as they were for the row correlations, although it typically produces average bicluster column correlation values greater than 0.5. As previously mentioned, OREO produces biclusters that are much larger than the other biclustering techniques, and it is interesting to note that the average correlation values over the columns for OREO are as large, and in some cases greater, than those found in the smaller biclusters produced by other methods. This observation is consistent when considering the column correlation as a function of the number of bicluster columns, as presented in Figures S.21, S.25, and S.29. It should be noted that ISA is observed to produce biclusters of substantially greater column correlation than row correlation (compare Figures S.18, S.22, and S.26 with Figures S.20, S.24, and S.28). However, this is a mathematical artifact since ISA produces biclusters that typically contain less than 5 columns, as shown in Figures S.21, S.25, and S.29, which presents the average column correlation as a function of the number of bicluster columns. The column correlations for the biclusters produced by SAMBA are of varying quality and have a high variation throughout the data sets. One should note that although the bicluster areas for SAMBA and ISA are generally consistent, SAMBA consistently has more columns per bicluster than ISA, which implies that ISA typically contains more rows per bicluster than SAMBA (this is confirmed in Figures S.19, S.23, and S.27). Cheng and Church's algorithm yields biclusters whose column correlations are of higher value than its row correlations; note that this observation is not a mathematical artifact of having a small number of columns per bicluster, as it was with ISA.

### Case Study 6: Yeast Segregant Gene Expression Data

The last data set used to test the proposed methodology is comprised of expression data for 6216 genes subject to 131 stress conditions [50]. Solving such a large-scale data set to optimality is a challenging task. OREO was able to optimally re-order the rows and columns of the matrix according to the objective function defined in Eq. 3 in approximately 19 hours of wall-clock time. Hierarchical clustering [4] and nsNMF [30] were also applied to re-order the experiments and genes of this data set and the hierarchical clustering leaves were also optimally re-ordered using TreeArrange [41]. The cophenetic correlation coefficient was computed for nsNMF [30] for factors *k *= 2 through 12 and it was found that a rank of four factors resulted in the highest coefficient. We also applied the biclustering algorithms BiMax and ISA to this data set and neither method was able identify significant biclusters for a variety of search parameters. To assess the biological significance of the re-ordered genes over all conditions, we examined biological processes from a curated gene ontology network for *S. cerevisiae *[51]. To evaluate the biological significance for neighboring genes, we evaluated the average enrichment for each of the 130 gene ontology terms over all possible neighborhoods of size L genes in the final ordering. For a specific neighborhood of size L genes, the biological process with the greatest enrichment is defined as the process with the maximum value according to the expression in Eq. 1.

(1) $$\text{Enrichment of process k}=\frac{(NG_{L}^{k}-1)/L}{NG^{k}/NG}$$

**Figure 5:**
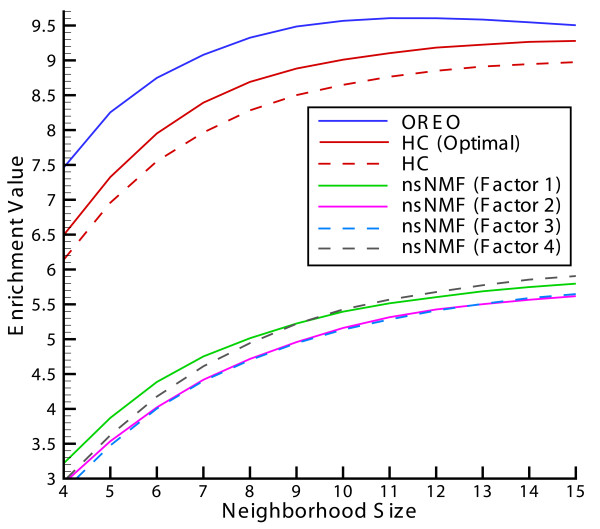
**Fold enrichment for re-ordered yeast segregant gene expression data**. Enrichment of genes sharing the same biological process annotation within different size neighborhoods for re-orderings by OREO, hierarchical clustering (HC), hierarchical clustering after optimal leaf ordering (HC (Optimal)), and nsNMF after sorting on the first, second, third and fourth basis conditions (nsNMF (Factor 1), nsNMF (Factor 2), nsNMF (Factor 3), nsNMF (Factor 4), respectively). For the definition of enrichment values, see Eq. 1 in text.

Where $NG_{L}^{k}$ denotes the number of genes in a neighborhood of size *L *for process *k*, *NG*^*k *^denotes the number of genes for process *k *in the entire experiment, and *NG *represents the total number of genes in the experiment. The term in the numerator in Eq. 1 represents the frequency of genes annotated with a given process, k, over the total number of genes considered, L. The frequency is adjusted for neighborhoods that have poor or random enrichment by subtracting one from the gene frequency. This enrichment is normalized by the term in the denominator, which is the fraction of the total number of genes annotated to process k in the experiment. This form of enrichment was applied to the re-ordered genes in order to fairly represent the contributions of interesting biological processes that are annotated to only a small subset of genes [see Additional file 1 for discussion]. Eq. 1 is applied for every process over all possible neighborhoods of genes, where the initial neighborhood of genes is comprised of genes of 1 though L in the final ordering and this neighborhood window is incremented by one gene (i.e., the next neighborhood contains genes 2 through L+1) until the last gene in the final ordering has been reached. The enrichment values in Eq. 1 are then averaged over the total number of neighborhoods considered. This process is repeated for several gene neighborhood sizes in the range of 4 to 15 genes and the results comparing our method to hierarchical clustering are shown in Figure 5.

One can see from Figure 5 that OREO achieves about 13 percent improvement in enrichment on average over the grouping of genes provided by hierarchical clustering. Although the enrichment for hierarchical clustering increases about 6 percent on average after applying TreeArrange to optimally re-order the leaves, it is still notably less than that provided by OREO. It is also shown in Figure 5 that the orderings provided by nsNMF after 50 runs when sorting the genes by either of the four basis conditions does not result in significant enrichment values.

The increased enrichment indicates that genes which are annotated to similar biological processes are arranged closer relative to one another in the final arrangement provided by OREO than for hierarchical clustering, with and without optimal leaf ordering, and nsNMF. When examining the individual process contributions to the average enrichment, we observed that electron transport, translation, hydrogen transport, ribosomal biogenesis/assembly and rRNA metabolism were the largest contributors to the overall enrichment value for both OREO and hierarchical clustering. Other biological processes of similar enrichment magnitudes for OREO include sulfur metabolism, aldehyde metabolism, carbohydrate transport, and mitochondrial transport, suggesting a better clustering of these genes. The deviations from optimality for the re-orderings provided by hierarchical clustering, with and without optimal leaf ordering, are presented in Table 1. We also present the results of our method for another data set consisting of an aggregation of experiments on budding yeast [4] [see Additional file 5].

## Conclusion

A rigorous method for biclustering based on the optimal re-ordering of dense data matrices, OREO, was presented in this article. The re-ordering of the rows and columns can be accomplished via either a network flow model or a traveling salesman problem representation, where the network flow model can be extended to include more than pairwise interactions. This iterative approach uses cluster boundaries in one dimension to define submatrices that are then optimally re-ordered in the other dimension to generate biclusters. Several different objective functions can be used to quantify the degree of similarity between adjacent rows and columns in the final arrangement and the selection of the appropriate metric is left as an option to the user. We compared the results of our method with several clustering and biclustering methods for (a) metabolite concentration data, (b) an image matrix, (c) synthetic data with implanted biclusters, and gene expression data for (d) colon cancer data, (e) breast cancer data, and (f) yeast segregant data. For each of these data sets, our method provides a closer grouping of related metabolites and annotated genes than the other clustering and biclustering algorithms, which suggests that the optimal re-ordering has distinct advantages over a local re-ordering and the simplifying assumptions of biclustering methods. It was also shown that OREO has the ability to separate objects into distinct groups, as was illustrated with the separation of the starvation conditions in the metabolite concentration data and the separation of samples in the colon and breast cancer data sets.

## Methods

In this section, we present the components of the mathematical model: (1) the variables, (2) the objective functions used to quantify pairwise similarity, and (3) two problem formulations which provide the optimal rearrangement of rows and columns, namely (a) a network flow model and (b) a traveling salesman (TSP) model. We then present a method for identifying cluster boundaries and iteratively biclustering submatrices via optimal re-ordering of submatrices. In the future, we plan to make this biclustering framework available to the academic community as a web-based tool.

### Variable Definitions

The index pair (*i*, *j*) corresponds to a specific row *i *and column *j *of a matrix, where the value of this pair is denoted as *a*_*i*,*j*_. The cardinality (or in this case, the dimension) of the rows and columns of the matrix will be represented as |*I*| and |*J*|, respectively. For the sake of brevity in this section and the remainder of the article, we present the terminology and mathematical model only for the rows of the matrix, but an analogous representation follows for the columns. We define the rows *i *and *i' *to be adjacent in the *final *arrangement of the matrix, where row *i' *is directly below row *i*. The final ordering of adjacent rows is represented via the following binary 0–1 variables.

$$y_{i,i^{\prime }}^{row}=\{\begin{array}{ll} 1, & \text{if row i is adjacent and above}\\ & \text{row i' in the final ordering}\\ 0, & \text{otherwise} \end{array}$$

For instance, if the binary variable *y*_8,3 _is equal to one then row 8 is immediately above row 3 in the final arrangement of the matrix. The assignment of *y*_8,3 _= 0 implies that row 8 is *not *immediately above row 3 in the final arrangement, but does not provide any additional information regarding the final positions of rows 8 and 3 in the matrix.

### Objective Function

The first stage of the proposed method optimally rearranges the rows and columns of a data matrix according to a given metric of similarity, which is left to the user to specify. In this section, we present common expressions that can be used for quantifying the similarity between two rows of a matrix. An intuitive metric of similarity is to minimize the relative difference in value for adjacent rows of a matrix, as presented in Eq. 2.

(2) $$\sum_{i}\sum_{i^{\prime }}\sum_{j}y_{i,i^{\prime }}^{row}\cdot \left|a_{i,j}-a_{i^{\prime },j}\right|$$

The emphasis can be placed on penalizing specifically large differences in value by squaring the difference in value between two adjacent rows and columns, as shown in Eq. 3.

(3) $$\sum_{i}\sum_{i^{\prime }}\sum_{j}y_{i,i^{\prime }}^{row}\cdot {\left(a_{i,j}-a_{i^{\prime },j}\right)}^{2}$$

A metric similar to the root-mean squared deviation of values can also be used to guide the rearrangement of the matrix, as shown in Eq. 4.

(4) $$\sum_{i}\sum_{i^{\prime }}y_{i,i^{\prime }}^{row}\cdot \sqrt{\frac{\sum_{j}{\left(a_{i,j}-a_{i^{\prime },j}\right)}^{2}}{\left|J\right|}}$$

The aforementioned objective functions can be tailored to exploit physical trends in the data set. For instance, suppose it is known a priori that the values of the data are monotonic when arranged in a particular order and that this final configuration is desirable. Then the terms in any of these objective functions could be easily restricted to include only those rows that violate such a monotonicity trend (i.e., *a*_*i*,*j *_> *a*_*i'*,*j*_). It is should be noted here that this monotonicity criterion is different than that of the biclustering algorithm OPSM [31]. Where OPSM is searching for the largest order-preserving submatrices for which the expression levels of all genes induce the same linear ordering across a subset of columns, the approach presented here would *allow *for monotonicity violations but penalize their contributions in the objective function. This is accomplished by only including the cost of placing elements *i *and *i' *adjacent in the final ordering if they violate the imposed monotonicity trend.

It should be noted that the objective functions defined in Eqs. 2 through 4 are symmetric, whereas incorporating monotonicity into these expressions introduces asymmetry. Each of these proposed metrics can result in distinctly different permutations of the final rearranged matrix. The objective functions presented in this section are typically used, however the model is not limited to these forms.

### Network Flow Model

A network flow model [52-57] is adequate for solving small and medium-sized problems and can be extended to incorporate more than pairwise comparisons. Note that the objective functions introduced in the previous section are independent of how the rows are physically permuted. The final ordering of the row permutations can be represented as a directed acyclic graph, where an edge connects two rows if these rows are *adjacent *in the final ordering.

As previously mentioned, the binary variables $y_{i,i^{\prime }}^{row}$ represent the assignment of a neighboring row *i' *directly below row *i *in the final arrangement. In network flow terminology, we say that the binary variable $y_{i,i^{\prime }}^{row}$ represents the existence of the edge between rows *i *and *i'*. We introduce another set of binary variables, $y\text{\_}source_{i}^{row}$ and $y\text{\_}sink_{i}^{row}$, to indicate which rows are assigned at the top and bottom of the final rearranged matrix, respectively.

$$\begin{array}{c} y\text{\_}source_{i}^{row}=\{\begin{array}{ll} 1, & \text{if row i is the top-most}\\ & \text{row in the final ordering}\\ 0, & \text{otherwise} \end{array}\\ y\text{\_}sink_{i}^{row}=\{\begin{array}{ll} 1, & \text{if row i is the bottom-most}\\ & \text{row in the final ordering}\\ 0, & \text{otherwise} \end{array} \end{array}$$

The flows values assigned to the edges connecting the rows are continuous variables denoted by $f_{i,i^{\prime }}^{row}$ These flows start from a fictitious source row and end at a fictitious sink row.

$$\begin{array}{lll} f_{i,i^{\prime }}^{row} & \equiv & \begin{array}{l} \text{the flow from row }i\\ \text{to row }i^{\prime } \end{array}\\ f\text{\_}source_{i}^{row} & \equiv & \begin{array}{l} \text{the flow entering}\\ \text{the source row }i \end{array}\\ f\text{\_}sink_{i}^{row} & \equiv & \begin{array}{l} \text{the flow leaving}\\ \text{the sink row }i \end{array} \end{array}$$

It should be noted that the variables $y_{i,i}^{row}$ and $f_{i,i}^{row}$ are zero since row *i *can never be adjacent to *itself*.

The physical act of connecting two rows by an edge (i.e., putting two rows adjacent to one another in the final arrangement) is modeled via the following constraint equations.

(5) $$\begin{array}{cc} \sum_{i^{\prime }\neq i}y_{i^{\prime },i}^{row}+y\text{\_}source_{i}^{row}=1 & \forall i \end{array}$$

(6) $$\begin{array}{cc} \sum_{i^{\prime }\neq i}y_{i,i^{\prime }}^{row}+y\text{\_}sink_{i}^{row}=1 & \forall i \end{array}$$

These constraints enforce that each row, *i*, has only one neighboring row above it (or is the top-most row) and only one neighboring row below it (or is the bottom-most row) in the final arrangement, respectively. The next two constraints ensure that only one top-most (source) row and only one bottom-most (sink) row should be assigned in the final matrix.

(7) $$\sum_{i}y\text{\_}source_{i}^{row}=1$$

(8) $$\sum_{i}y\text{\_}sink_{i}^{row}=1$$

The set of constraints defined by Eqs. 5 through 8 are sufficient for assigning unique neighbors to every row. However, *cyclic *arrangements of the rows also satisfy these constraint equations (i.e., it is possible to have $y_{i,i^{\prime }}^{row}=y_{i^{\prime },i^{\prime \prime }}^{row}=y_{i^{\prime \prime },i}^{row}=10$, which results in a cyclic final ordering of *i*, *i'*, *i"*, *i*, ... etc.) To ensure that the final arrangement of the rows is acyclic, unique flow values are assigned to each edge, $y_{i,i^{\prime }}^{row}$, that connects rows *i *and *i'*. The value for the flow entering the source row (or top-most row) is defined to be the total number of rows (|*I*|) to indicate that this is the top-most row in the final arrangement.

(9) $$\begin{array}{cc} f\text{\_}source_{i}^{row}=\left|I\right|\cdot y\text{\_}source_{i}^{row} & \forall i \end{array}$$

Note that the above constraint, in conjunction with Eq. 7, ensures that only one source flow is assigned to an edge. Starting from this source row, each subsequent row in the final arrangement will have an entering flow value of |*I*| - 1, |*I*| - 2, and so on. This cascading property of the flow values will ensure a unique final ordering of the rows and eliminate cyclic arrangements. A flow conservation equation is used to model this cascading of the flows by requiring that the flow entering a row is exactly one unit greater than the flow leaving that row.

(10) $$\begin{array}{c} \sum_{i^{\prime }}(f_{i^{\prime },i}^{row}-f_{i,i^{\prime }}^{row})+f\text{\_}source_{i}^{row}\\ \begin{array}{cc} -f\text{\_}sink_{i}^{row}=1 & \forall i \end{array} \end{array}$$

Since we have defined the convention that $f\text{\_}source_{i}^{row}$ starts at |*I*|, then $f\text{\_}sink_{i}^{row}$ has a flow value of *zero *and thus can be eliminated from the above constraint.

Lastly, we can assign general upper and lower bounds for all flow values since a flow connecting two rows *i *and *i' *(i.e., $y_{i,i^{\prime }}^{row}$ = 1) can never be greater than |*I*| - 1 nor less than 1.

(11) $$\begin{array}{cc} f_{i,i^{\prime }}^{row}\leq (|I|-1)\cdot y_{i,i^{\prime }}^{row} & \forall (i,i^{\prime }) \end{array}$$

(12) $$\begin{array}{cc} f_{i,i^{\prime }}^{row}\geq y_{i,i^{\prime }}^{row} & \forall (i,i^{\prime }) \end{array}$$

These constraint equations also ensure that if rows *i *and *i' *are not connected by an edge (i.e., $y_{i,i^{\prime }}^{row}$ = 0) then no flow is assigned ($f_{i,i^{\prime }}^{row}$ = 0). The set of constraint equations (5)–(12) comprise the entire mathematical model necessary for performing the row and column permutations, which are guided by any of the aforementioned objective functions.

### TSP Model

The re-ordering of the rows and columns can also be modeled as a traveling salesman problem (TSP), which is one of the most well-studied problems in the area of combinatorial optimization. The problem objective is to visit a list of *N *cities and return to the starting city via the minimum cost route (often referred to as the optimal tour). Finding the best tour and guaranteeing its optimality remains challenging for large-scale problems. It has been pointed out that the row and column re-ordering problems can be solved as two independent traveling salesman problems [58].

In the TSP formulation, each row in the matrix is a vertex, *i *∈ |*I*|. The existence of an edge between rows *i *and *i' *is again represented by the binary variable $y_{i,i^{\prime }}^{row}$. For each edge there is an associated cost, *c*_*i*, *i' *_of "traveling" from row *i *to *i'. *Thus, the objective of the problem is to visit each row in the matrix only once via these edges while incurring the minimum total cost and the order in which these rows are visited denotes their final positions in the matrix. The problem definition requires that the tour start and end at the same row, so we introduce a dummy city to connect the top-most and bottom-most row in the final arrangement with edges that have zero cost. A formal definition of the problem is provided below.

(13) $$\min\sum_{i,i^{\prime }}c_{i,i^{\prime }}\cdot y_{i,i^{\prime }}^{row}$$

(14) $$\begin{array}{cc} \sum_{i^{\prime }}y_{i,i^{\prime }}^{row}=1 & \forall i \end{array}$$

(15) $$\begin{array}{cc} \sum_{i^{\prime }}y_{i^{\prime },i}^{row}=1 & \forall i \end{array}$$

The cost associated with traversing an edge, *c*_*i*, *i*'_, is computed using the aforementioned objective functions. As in the network flow model, cyclic tours satisfy Eqs. 14 and 15, thus additional constraints are required to eliminate these *subtours*. These constraints are efficiently incorporated into TSP solvers, such as Concorde [59], via cutting plane methods and are beyond the scope of this paper so will not be discussed here.

Although the idea of traveling implies moving from one row to the next, if the cost of traveling in either direction is the same for any row, then the problem is symmetric and only *undirected *edges between rows need to be considered. However, the objective functions that incorporate monotonicity violations are by definition asymmetric and require an asymmetric TSP formulation. The asymmetric traveling salesman problem can be recast as a symmetric traveling salesman problem by introducing a duplicate set of N rows and restricting the overall connectivity of edges. The details of how to perform such a transformation have been described elsewhere [60,61] and will not be presented here.

### Iterative Framework

The algorithm begins by optimally re-ordering a single dimension of the data matrix. Let us denote the dimension that is re-ordered as the columns and the dimension that is not re-ordered as the rows of the data matrix. For instance, in gene expression data the columns would correspond to the time series or set of conditions over which the expression level for the genes of interest (i.e., the rows) were measured. The objective function value for each pair-wise term between neighboring columns in the final ordering is evaluated and the median of these values is computed. That is, for each column *j *and *j *+ 1 in the *final *ordering, the median of each pairwise term of the objective function, *ϕ*(*a*_*i*,*j*_, *a*_*i*,*j*+1_), is computed, as shown in Eq. 16.

(16) *MEDIAN*_*i *_*ϕ*(*a*_*i*,*j*_, *a*_*i*,*j*+1_)

The median was selected as the evaluating metric since it is statistically less biased to outliers than the average. Cluster boundaries are defined to lie between those columns which have the *largest *median values (since the objective function is being minimized). In other words, the median is computed for all pairs *j *and *j *+ 1 in the final ordering and the top 10 percent of largest median values are selected as boundaries between the re-ordered columns. These cluster boundaries are used to partition the original matrix into several submatrices. The rows of each submatrix are then optimally re-ordered over their subset of columns and clusters in this dimension are again defined using the median value of the objective function between neighboring rows in the final ordering. The algorithmic steps for the iterative framework are presented below:

1. Optimally re-order a single dimension of the data matrix. This re-ordered dimension will be denoted as the columns.

2. Compute the median for each pair of neighboring columns in the final ordering using Eq. 16. Sort these values from highest to lowest; the largest median values define the cluster boundaries between the columns. Submatrices are defined by the columns that lie between these cluster boundaries.

3. Optimally re-order the rows of each submatrix and compute the cluster boundaries for the re-ordered rows analogous to step 2.

## Authors' contributions

PAD, CAF, and SRM developed and implemented the proposed mathematical models, conducted the numerical experiments and subsequent analysis, and drafted the manuscript. XJF, JDR, and HAR participated in the design of the study, interpretation of data, and helped to draft and revise the manuscript. All authors read and approved the final manuscript.

## Supplementary Material

Additional file 1 **This additional file contains all of the additional figures and results referenced in the article**. Click here for file

Additional file 2 **This text file contains the results for the biclustering algorithms Cheng and Church's, ISA, OPSM, BiMax, xMotif, and SAMBA for the Alon et. al colon cancer data set**. Click here for file

Additional file 3 **This Excel spreadsheet contains the complete ontology results for all biclustering algorithms using Onto-Express for the Alon et al. and van't Veer cancer data sets**. Click here for file

Additional file 4 **This text file contains the results for the biclustering algorithms Cheng and Church's, ISA, OPSM, BiMax, xMotif, and SAMBA for the van't Veer et al. breast cancer data set**. Click here for file

Additional file 5 **This text file contains the corresponding clusters for OREO described in the article for the Eisen et al. budding yeast data set (described in Additional file**1**).** Click here for file
