# Supplementary material for: Biclustering via optimal re-ordering of data matrices in systems biology: rigorous methods and comparative studies
Source: BMC Bioinformatics. 2008 Oct 27;9:458. doi: 10.1186/1471-2105-9-458 (PMC2605474; doi:10.1186/1471-2105-9-458)
Supplement: Additional file 1 — This additional file contains all of the additional figures and results referenced in the article. [file 1471-2105-9-458-S1.pdf]

Supplementary Material for Biclustering via Optimal Re-ordering of Data Matrices

Case Study 1: Biclustering Results for Metabolite Concentration Data

The optimally re-ordered metabolites for region A for OREO result in a dense grouping of the amino acids and biosynthetic intermediates under the conditions of nitrogen starvation in *S. cerevisiae.* One should note the almost monotonic behavior of the concentration profile, which groups the decreasing concentrations at the top and the increasing concentrations at the bottom of the matrix. The twelve amino acids glycine, asparigine, serine, alanine, methionine, threonine, histidine, aspartate, tryptophan, phenylalanine, isoleucine, and valine are found in a cluster of 26 metabolites. There is also a strong aggregation of the biosynthetic intermediates carbamoyl-aspartate, ornithine, dihydrooroate, N-acetyl-ornithine, IMP, cystathionine, and orotic acid in the first nine rows of the data matrix. This supports the observation that most biosynthetic intermediates decrease in concentration over all starvation conditions based on the hypothesis that the cells turn off de novo biosynthesis as an early, strong, and consistent response to nutrient deprivation [1]. One should also note that carbamoyl-aspartate and dihydrooroate, separated by only two positions in the final ordering, are both on the pyrimidine pathway [1].

Case Study 1: Results for Other Biclustering Methods for Metabolite Concentration Data

The results for Cheng and Church's Algorithm [2] reported a total of 10 biclusters. A bicluster of 30 metabolites contained 15 of the 18 metabolites assigned to the amino acid category [1] over various conditions related to carbon and nitrogen starvation in *E. coli* and *S. cerevisiae.* The longest consecutive ordering of amino acids within this bicluster are serine, methionine, threonine, glutamate, and alanine, which is exactly the same as that reported in the hierarchical clustering results. The majority of the other metabolites in this bicluster are biosynthetic intermediates. The remaining nine

biclusters do not result in any consistent grouping of related metabolites, although three biclusters contain only nitrogen starvation conditions and two biclusters contain only two nitrogen starvation conditions for *E. coli* and *S. cerevisiae.* In fact, one of the biclusters contains all of the nitrogen starvation conditions for *E. coli.* Only one bicluster was reported from the ISA algorithm [3]. The four metabolites in this bicluster are acetyl-coa, aspartate, ADP, and cAMP, under the conditions of carbon starvation in *E. coli.* Acetyl-coa is a TCA cycle compound and aspartate is categorized as a amino acid metabolite [1], so there is no direct relationship between the metabolites identified in this bicluster. The OPSM Algorithm [4] produced a bicluster that contained the amino acid metabolites valine, isoleucine, alanine, phosphoenolpyruvate, ATP, proline, asparigine, and glutamate under the conditions of nitrogen and carbon starvation in *S. cerevisiae* and *E. coli.* The other OPSM biclusters revealed little correlation among the metabolites grouped together. The BiMax Algorithm [5] resulted in several biclusters of increased concentration and many of the metabolites were assigned to at least three biclusters. However, little relation among the metabolites in the BiMax biclusters was found. The SAMBA Algorithm [6] was also applied to the metabolite concentration data and resulted in a total of three biclusters with little overlap of the metabolites. No apparent grouping of related metabolites was observed for these biclusters.

[Figure 1 about here.]

[Figure 2 about here.]

[Figure 3 about here.]

[Figure 4 about here.]

[Figure 5 about here.]

[Figure 6 about here.]

[Figure 7 about here.]

Case Study 2: Image Reconstruction (Lenna Image)

When optimally re-ordering the replicated data matrix using OREO, we see in Figure S.8 that we do recover a stretched version of the original image. In Figure S.8, we see that the Memetic algorithm [7] was also able to recover the elongated image, whereas the two hierarchical clustering methods and CLICK [8] could not. The agglomerative hierarchical clustering algorithm [9] recovers most of the image but misplaces an entire subsection near the top. The European Bioinformatics Initiative (EBI) hierarchical clustering algorithm [10] and CLICK partition the image into choppy subsections with no continuity between them.

[Figure 8 about here.]

In the original study [11], it was also shown that re-ordering over the 100 adjacent columns centered in the region of the eyes proved to be the most difficult task for all of the clustering methods. In Figure S.9 we present the results for all of the algorithms, where it can be seen that only OREO and the Memetic algorithm recover the original image. Note that in contrast to the previous examples, agglomerative hierarchical clustering performs significantly worse and partitions the original image into several subclusters, as do EBI hierarchical clustering algorithm and CLICK.

[Figure 9 about here.]

Case Study 3: Synthetic Data with Implanted Biclusters

In Figures S.10 through S.17 we present the average bicluster relevance and average module recovery for the results of OREO for the synthetic data set with implanted biclusters presented in [5].

[Figure 10 about here.]

[Figure 11 about here.]

[Figure 12 about here.]

[Figure 13 about here.]

[Figure 14 about here.]

[Figure 15 about here.]

[Figure 16 about here.]

[Figure 17 about here.]

Cancer Study 4: Colon Cancer Data

The exact classification of the tissues are presented in Table S.1 for the method presented in Alon et al. and OREO for colon cancer data set [12].

[Table 1 about here.]

Case Studies 1, 4, and 5: Plots of Correlation Values for Biclustering Results

In Figures S.18 through S.29, we present the average correlation among the rows and columns as a function of (1) the bicluster area (the number of rows times the number of columns in a bicluster), (2) the number of rows per bicluster, and (3) the number of columns per bicluster for the (a) metabolite concentration data, (b) colon cancer data, and (c) breast cancer data sets. Discussions for these figures are provided in the main text of the article.

[Figure 18 about here.]

[Figure 19 about here.]

[Figure 20 about here.]

[Figure 21 about here.]

[Figure 22 about here.]

[Figure 23 about here.]

[Figure 24 about here.]

[Figure 25 about here.]

[Figure 26 about here.]

[Figure 27 about here.]

[Figure 28 about here.]

[Figure 29 about here.]

Case Study 6: Curated Gene Ontology Network and nsNMF Results for Yeast Segregant Gene Expression Data

We examined biological processes from a curated gene ontology network for *S. cerevisiae* as judged by a panel of expert scientists [13]. From this curated data set of 295 biological processes, we extracted those that are annotated to at least ten or more genes. This screening process resulted in a total 130 biological processes for subsequent analysis. Out of the 6216 genes analyzed in the experiment, 4152 are annotated to at least one of these 130 selected processes. For the remaining genes that were not annotated to any of the 130 processes, we created an "un-annotated" process to analyze their placements relative to the annotated genes.

The metric presented in Equation 16 in the text avoids bias towards processes that are annotated to a large number of genes in the experiment. For instance, processes such as translation and ribosomal biogenesis and assembly that are annotated to 683 and 331 genes, respectively, are more likely to be found grouped together random than processes that are annotated to a small subset of genes, like aldehyde metabolism (annotated to 21 genes) and carbohydrate transport (annotated to 36 genes). Equation 16 effectively accomplishes the task of properly weighting the enrichment contributions of processes annotated to only a small number of genes but are considered important [13] like aldehyde metabolism and carbohydrate transport.

We also queried the top 200 genes from nsNMF after sorting on the basis conditions using the Gene Ontology Term Finder (http://db.yeastgenome.org/cgi-bin/-GO/goTermFinder.pl) to examine if enrichment was achieved for specific biological processes. Sorting on factor 1 resulted in a significant grouping of genes annotated to the energy reserve metabolic process (P-value = 3.81×10^{-13}). The genes based on sorting on factor 2 were significantly annotated to the RNA-mediated transposition process (P-value = 1.50×10^{-18}) and the richest annotation of genes was found from sorting on factor 3 for the amino acid metabolic process (P-value = 8.04×10^{-22}). Sorting the genes based on the fourth factor resulted in a significant grouping that was annotated to the telomere maintenance via recombination process (P-value = 1.40×10^{-4}). This indicates that although the orderings of genes provided by the individual factors for nsNMF do not result in a significant overall enrichment (presented in Figure 4), they do produce significant groupings of genes related to important biological processes.

Additional Gene Expression Data Set: Budding Yeast Gene Expression Data

Another data set commonly studied in the literature is an aggregation of experiments on budding yeast, consisting of 6,222 genes and 80 experimental conditions [9]. In the original study, hierarchical clustering was applied to the 2,4567 genes that had a functional annotation in the Saccharomyces Genome Database. It was shown that genes of similar function cluster together, and in particular genes encoding: (a) ribosomal proteins, (b) respiration, (c) ATP synthesis and oxidative phosphorylation, (d) components of the proteasome, (e) DNA replication, (f) the tricarboxylic acid cycle and oxidative phosphorylation, (g) glycolytic enzymes, and (h) protein synthesis [9].

This data set was analyzed in another study [11] by a Memetic and hierarchical clustering algorithm, where several clusters annotated to specific functional groups were presented. Specifically, the Memetic and hierarchical clustering algorithm were applied to the 3,222 genes without missing values and the clusters that were reported

were annotated to the functions "protein synthesis" (similar to (h)) , "protein degradation" (similar to (d)), "ATP synthesis + oxidative phosphorylation + TCA cycle" (combinations of (c) and (f)), and "sterol metabolism". We optimally reordered the 3,222 genes using OREO (results available as another file in Supplementary Material) and compared our results to the previous findings [9, 11].

We observed a large clustering of genes encoding "protein synthesis" (78 genes), which is consistent with all the other methods, as each was able to find a clustering of at least 70 of these terms. The most variability in the results was found in the clustering of the smaller functional groups. Note that the functional groups for the clusters presented for the Memetic algorithm [11] are very similar to the ones described in the original study (namely "protein degradation" and "ATP synthesis + oxidative phosphorylation + TCA cycle") . Our method also uncovers clusters enriched in these molecular functions, where our clusters for "oxidative phosporylation" are separated into "ATP + oxidative phosphorylation" (8 genes) and "TCA + oxidative phosphorylation" (7 genes), which is consistent with the findings of the original study [9]. The hierarchical clustering algorithm also produced this partitioning of the genes encoding "oxidative phosphorylation". Another consistency with the original study is that our cluster of genes associated with the function "protein degradation" (12 genes) was found to be rich in those genes encoding the components of the proteasome. The "protein degradation" cluster for the Memetic algorithm was found to have a similar number of proteasome genes (14 genes), whereas the hierachical clustering algorithm was able to cluster together 18 proteasome genes [7].

The only functional group reported by [11] that was not presented in the original study was "sterol metabolism", where the Memetic algorithm, hierarchical clustering algorithm, and OREO reported clusters of 7, 4, and 5 genes annotated to this function, respectively. Several other clusters were found by OREO that were consistent with the findings in the original study, such as "glycolysis" (8 genes), "DNA replication" (5 genes), and "spindle pole body assembly and function" (8 genes). We also analyzed our results for clusters associated with more interesting molecular functions

not reported in the earlier studies. For instance, we discovered a tight cluster containing all 6 histone genes in consecutive ordering, which is similar to the histone cluster presented in the original study, except our cluster also contains Histone H1. There is another interesting cluster containing 7 genes associated with the functions of MRNA and TRNA splicing, processing, and exporting. When searching the entire set of genes, there are only 14 functional annotations associated with membrane proteins and our method produces a tight cluster consisting of 4 genes corresponding to membrane protein functions, 3 of which are vacuolar proteins. Of the 8 genes associated with peroxisome biogenesis, we found a cluster of 4 within a span of 13 genes, whereas these genes were scattered in the original hierarchical clustering results. There are also two clusters in which genes associated with asparagine utilization (4 genes in cluster, 7 in total data set) and allantoin utilization (3 genes in cluster, 4 in total data set) are consecutively ordered. Lastly, there is an interesting cluster of 6 genes associated with various amino acid biogenesis functions (arginine, isoleucine and valine, threonine and methionine, and histidine biosynthesis) that were not found when examining the results of the original study.

Table S.2 - Ontology results for Alon et al. colon cancer data for all biclustering algorithms

Ontology results obtained for the biclustering algorithms Cheng and Church, ISA, OPSM, xMotif, OREO, nsNMF, and SAMBA using Onto-Express for the Alon et al. colon cancer data set. A hypergeometric distribution was used and the reference array was specified to be the 2,000 genes analyzed. Biclusters are only reported for each method if they have a p-value less than 0.01.

Cheng and Church's Biclusters

Cluster No.	No. genes	molecular function	biological process
2	72	peroxiredoxin activity	repsonse to oxidative stress
3	11	deoxyguanosine kinase activity	guanosine metabolic process
4	6	tyrosine 3-monooxygenase activity	catecholamine biosynthetic process
5	5	single-stranded RNA binding	RNA destabilization
6	3	GDP-dissociation inhibitor activity	G-protein signaling
10	3	fucosyltransferase activity	L-fucose catabolic process

ISA Biclusters

Cluster No.	No. genes	molecular function	biological process
1	128	transmembrane receptor protein tyrosine phosphatase activity	negative regulation of protein kinase activity
2	111	cAMP-dependent protein kinase activity	transcription
3	98	threonine endopeptidase activity	ubiquitin-dependent protein catabolic process
4	130	vinculin binding transforming growth factor beta	receptor signaling pathway
5	97	threonine endopeptidase activity	ubiquitin-dependent protein catabolic process
6	96	nucleotide binding	ubiquitin-dependent protein catabolic process
7	81	cadmium ion binding	none
8	148	ligase activity	regulation of small GTPase mediated signal transduction
9	147	ligase activity	regulation of small GTPase mediated signal transduction
10	87	transcription activator	ubiquitin-dependent protein catabolic process

OPSM Biclusters

Cluster No.	No. genes	molecular function	biological process
2	3	mRNA 3'-UTR binding	cell differentiation
3	4	formlytetrahydrofolate dehydrogenase activity	10-formyltetrahydrofolate catabolic process
4	6	phosphatase binding	cell-cell adhesion
5	11	3'-5-' exonuclease activity	regulation of cell redox homeostasis
6	25	none	mRNA catabolic process
7	48	DNA binding	regulation of small GTPase mediated signal transduction
8	94	nucleotide binding	protein amino acid phosphorylation
9	226	ATP binding	protein amino acid phosphorylation
10	456	actin binding	Wnt receptor signaling pathway

xMotif Biclusters

Cluster No.	No. genes	molecular function	biological process
1	562	nucleotide binding	cell migration
2	505	none	cell migration
3	494	none	immune response
4	491	identical protein binding	none
5	480	extracellular matrix structural constituent	translation
6	473	none	notch signaling pathway
7	473	protein serine/threonine kinase activity	cell migration
8	471	receptor binding	immune response
9	467	nucleotide binding	immune response
10	467	none	immune response
11	467	none	immune response
12	462	none	immune response

OREO Biclusters

Cluster No.	No. genes	molecular function	biological process
1	97	structural constituent of ribosome	translation
2	41	structural constituent of ribosome	translation
3	114	structural constituent of ribosome	translation
4	69	structural constituent of ribosome	translation
5	153	structural constituent of ribosome	translation
7	575	structural molecule activity	establishment and/or maintenance of cell polarity
8	1687	structural constituent of ribosome	translation
9	289	structural constituent of ribosome	translation

nsNMF Biclusters

Cluster No.	No. genes	molecular function	biological process
Factor 1	100	structural constituent of ribosome	translation
Factor 2	100	structural constituent of ribosome	antigen processing and presentation

SAMBA Biclusters

Cluster No.	No. genes	molecular function	biological process
15	16	zinc ion binding	regulation of cell growth
18	10	receptor activity	axon guidance
25	17	structural constituent of ribosome	translation
26	16	structural constituent of ribosome	translation
27	22	structural constituent of ribosome	translation
28	22	structural constituent of ribosome	translation
29	15	structural constituent of ribosome	translation
30	16	structural constituent of ribosome	translation
31	16	structural constituent of ribosome	translation
32	16	structural constituent of ribosome	translation
33	22	structural constituent of ribosome	translation
34	28	structural constituent of ribosome	translation
35	20	structural constituent of ribosome	translation
36	19	structural constituent of ribosome	translation
37	20	structural constituent of ribosome	translation

Table S.3 - Ontology results for van't Veer et al. breast cancer data for all biclustering algorithms

Ontology results obtained for the biclustering algorithms Cheng and Church, ISA, OPSM, xMotif, OREO, nsNMF, and SAMBA using Onto-Express for the van't Veer et al. breast cancer data set. A hypergeometric distribution was used and the reference array was specified to be the 5,000 genes analyzed. Biclusters are only reported for each method if they have a p-value less than 0.01. Note that xMotif was unable to produce any biclusters as the number of samples (98) was too large for the method.

Cheng and Church's Biclusters

Cluster No.	No. genes	molecular function	biological process
2	44	4-alpha-glucanotransferase	meiotic spindle organization and biogenesis
4	37	aminomethyltransferase activity	C21-steroid hormone metabolic process
5	26	acetyl-CoA transporter activity	positive regulation of endothelial cell differentitation
6	34	none	regulation of cytoskeleton organization and biogenesis
7	34	glycine dehydrogenase activity	generation of precursor metabolites and energy
8	32	ceramide kinase activity	icosanoid biosynthetic process
9	30	3-galactosly-N-acetylglucosaminide 4-alpha-L-fucosyltransferase activity	axial mesoderm morphogenesis
10	31	cytokine activity	macroautophagy

ISA Biclusters

Cluster No.	No. genes	molecular function	biological process
1	207	heparin binding	cell motillity
2	206	heparin binding	Notch signaling pathway
3	206	structural constituent of ribosome	chromosome organization and biogenesis
4	203	heparin binding	angiogenesis
5	215	GDP binding	G-protein signaling, adenylate cyclase inhibiting pathway
6	210	vitamin D receptor binding	chromosome organization and biogenesis
7	207	oxygen binding	oxygen transport
8	207	metabotropic glutamate, GABA-B like receptor activity	nucleosome assembly
9	203	vitamin D receptor binding	C21-steroid hormone biosynthetic process
10	197	DNA binding	chromosome organization and biogenesis (sensu Eukaryota)
11	196	DNA binding	chromosome organization and biogenesis (sensu Eukaryota)
12	259	mRNA binding	DNA damage response, signal transduction by p53 class mediator
13	192	none	zinc ion transport
14	244	ErbB-3 class receptor binding	tricarboxylic acid cycle
15	180	metal ion binding	very-long-chain fatty acid metabolic process

OPSM Biclusters

Cluster No.	No. genes	molecular function	biological process
1	2	receptor signaling protein activity	none
2	3	MHC class II protein binding	regulation of macrophage activation
3	5	interleukin-3 receptor activity	signal transduction
4	10	chemoattractant activity	regulation of progression through S phase
5	16	interleukin-10 receptor activity	signal transduction
6	35	C-C chemokine receptor activity	immune response
7	59	guanyl-nucleotide exchange factor activity	immune response
8	115	C-C chemokine receptor activity	immune response
9	221	chemokine activity	immune response
10	455	MHC class I receptor activity	immune response
11	995	chemokine activity	immune response
12	2007	transmembrane receptor activity	immune response

OREO Biclusters

Cluster No.	No. genes	molecular function	biological process
1	116	proteasome activator activity	immune response
2	248	isopentenyl-diphosphate delta-isomerase activity	positive regulation of alpha-beta T cell proliferation
5	1027	MHC class II receptor activity	antigen processing and presentation of peptide or polysaccharide antigen via MHC class II
hline 6	31	chemokine activity	immune response
8	28	MHC class II receptor activity	antigen processing and presentation of peptide or polysaccharide antigen via MHC class II
hline 12	380	structural constituent of ribosome	glycolysis
14	271	structural constituent of ribosome	translation
17	23	MHC class I rececptor activity	antigen processing and presentation of peptide antigen via MHC class I
33	480	3-chloroallyl aldehyde dehydrogenase activity	intracellular signaling cascade
41	584	structural constituent of ribosome	translation
39	807	MHC class II receptor activity	antigen processing and presentation of peptide or polysaccharide antigen via MHC class II

nsNMF Biclusters

Cluster No.	No. genes	molecular function	biological process
Factor 1	200	sugar binding	immune response
Factor 2	200	symporter activity	fatty acid biosynthetic process

SAMBA Biclusters

Cluster No.	No. genes	molecular function	biological process
20	78	extracellular matrix structural constituent	phosphate transport
24	76	aminoacyl-tRNA ligase activity	tRNA aminoacylation for protein translation
27	87	chemokine activity	immune response
3	124	chemokine activity	immune response
30	137	wide-spectrum protease inhibitor activity	homophilic cell adhesion
31	146	serine-type endopeptidase activity	homophilic cell adhesion
34	77	chemokine activity	immune response
36	34	double-stranded DNA specific 5'-3' exodeoxyribonuclease activity	cell division
77	88	extracellular matrix structural constituent	phosphate transport
78	34	structural molecule activity	epidermis development
82	35	steroid binding	very-long-chain fatty acid metabolic process
84	39	unspecific monooxygenase activity	electron transport
87	135	unspecific monooxygenase activity	pigmentation
93	51	unspecific monooxygenase activity	electron transport
94	45	unspecific monooxygenase activity	electron transport

References

[1] M. J. Brauer, J. Yuan, B. Bennett, W. Lu, E. Kimball, D. Bostein, and J.D. Rabinowitz. Conservation of the metabolomic response to starvation across two divergent microbes. *Proc. Natl. Acad. Sci.*, 103:19302–19307, 2006.

[2] Y. Cheng and G.M. Church. Biclustering of expression data. *Proc. ISMB 2000*, pages 93–103, 2000.

[3] J. Ihmels, G. Friedlander, S. Bergmann, O. Sarig, Y. Ziv, and N. Barkai. Revealing modular organization in the yeast transcriptional network. *Nat. Genet.*, 31:370–377, 2002.

[4] A. Ben-Dor, B. Chor, R.Karp, and Z.Yakhini. Discovering local structure in gene expression data: The order-preserving submatrix problem. In *RECOMB*, 2002.

[5] A. Prelic, S. Bleuler, P. Zimmermann, A. Wille, P. Buhlmann, W. Gruissem, L. Hennig, L. Thiele, and E. Zitzler. A systematic comparison and evaluation of biclustering methods for gene expression data. *Bioinformatics*, 22(9):1122–1129, 2006.

[6] A. Tanay, R. Sharan, and R. Shamir. Discovering statistically significant biclusters in gene expression data. *Bioinformatics*, 18:S136–S144, 2002.

[7] C. Cotta, A. Mendes, V. Garcia, P. Franca, and P. Moscato. Applying Memetic algorithms to the analysis of microarray data. In *Lecture notes in computer science*, pages 22–32. Springer-Verlag Berlin, 2003.

[8] R. Shamir and R. Sharan. Algorithmic approaches to clustering gene expression data. In T. Jiang, T. Smith, Y. Xu, and M. Zhang, editors, *Cuurent Topics in Computational Biology*, pages 269–299. MIT Press, 2002.

[9] M. B. Eisen, P. T. Spellman, P. O. Brown, and D. Botstein. Cluster analysis and display of genome-wide expression patterns. *Proc. Natl. Acad. Sci.*, 95:14863–14868, 1998.

[10] J. Vilo, M. Kapushesky, P. Kemmeren, U. Sarkans, and A. Brazma. *Expression Profiler*. Springer-Valeg, 2003.

[11] P. Moscato, A. Mendes, and R. Berretta. Benchmarking a Memetic algorithm for ordering microarray data. *Biosystems*, 88(1):56–75, 2007.

[12] U. Alon, N. Barkai, D.A. Notterman, K. Gish, S. Ybarra, D. Mack, and A.J. Levine. Broad patterns of gene expression revealed by clustering analysis of tumor and normal colon tissues probed by oligonucleotide arrays. *Proc. Natl. Acad. Sci.*, 96:6745–6750, 1999.

[13] T. Reguly, A. Breitkreutz, L. Boucher, B. Breitkreutz, G. Hon, C.L. Myers, A. Parsons, H. Friesen, R. Oughtred, A. Tong, Y. Ho, D. Botstein, B. Andrews, C. Boone, O.G. Troyanskya, T. Ideker, K. Dolinski, N.N. Batada, and M. Tyers. Comprehensive curation and analysis of global interaction networks in saccharomyces cerevisiae. *Journal of Biology*, 5(11):1–38, 2006.

List of Figures

S.1 Biclustering Results for Cheng and Church's Algorithm 21
S.2 Biclustering Results for Cheng and Church's Algorithm: Enlarged . . 22
S.3 Biclustering Results for Cheng and Church's Algorithm: Enlarged . . 23
S.4 Biclustering Results for ISA Algorithm 24
S.5 Biclustering Results for OPSM Algorithm 25
S.6 Biclustering Results for BiMax Algorithm 26
S.7 Biclustering Results for SAMBA Algorithm 27
S.8 The clustering results for OREO, a Memetic algorithm, EBI hierarchical clustering, agglomerative hierarchical clustering, and CLICK for the Lenna image replicated row-wise ten times. Note that the only two methods able to recover the correct ordering are OREO and the Memetic algorithm. 28
S.9 The clustering results for OREO, a Memetic algorithm, EBI hierarchical clustering, agglomerative hierarchical clustering, and CLICK for the partial Lenna image replicated row-wise ten times. Note that the only two methods able to recover the correct ordering are OREO and the Memetic algorithm. 29
S.10 Average bicluster relevance for OREO for constant, non-overlapping biclusters. 30
S.11 Average module recovery for OREO for constant, non-overlapping biclusters. 31
S.12 Average bicluster relevance for OREO for additive, non-overlapping biclusters. 32
S.13 Average module recovery for OREO for additive, non-overlapping biclusters. 33
S.14 Average bicluster relevance for OREO for constant, overlapping biclusters. 34
S.15 Average module recovery for OREO for constant, overlapping biclusters. 35
S.16 Average bicluster relevance for OREO for additive, overlapping biclusters. 36
S.17 Average module recovery for OREO for additive, overlapping biclusters. 37
S.18 Absolute value of average correlation values among rows for metabolite concentration data as a function of bicluster area. 38
S.19 Absolute value of average correlation values among rows for metabolite concentration data as a function of bicluster row size. 39
S.20 Absolute value of average correlation values among columns for metabolite concentration data as a function of bicluster area. 40
S.21 Absolute value of average correlation values among columns for metabolite concentration data as a function of bicluster column size. 41
S.22 Absolute value of average correlation values among rows for colon cancer data as a function of bicluster area. 42

S.23 Absolute value of average correlation values among rows for colon cancer data as a function of bicluster row size. 43
S.24 Absolute value of average correlation values among columns for colon cancer data as a function of bicluster area. 44
S.25 Absolute value of average correlation values among columns for colon cancer data as a function of bicluster column size. 45
S.26 Absolute value of average correlation values among rows for breast cancer data as a function of bicluster area. 46
S.27 Absolute value of average correlation values among rows for breast cancer data as a function of bicluster row size. 47
S.28 Absolute value of average correlation values among columns for breast cancer data as a function of bicluster area. 48
S.29 Absolute value of average correlation values among columns for breast cancer data as a function of bicluster column size. 49

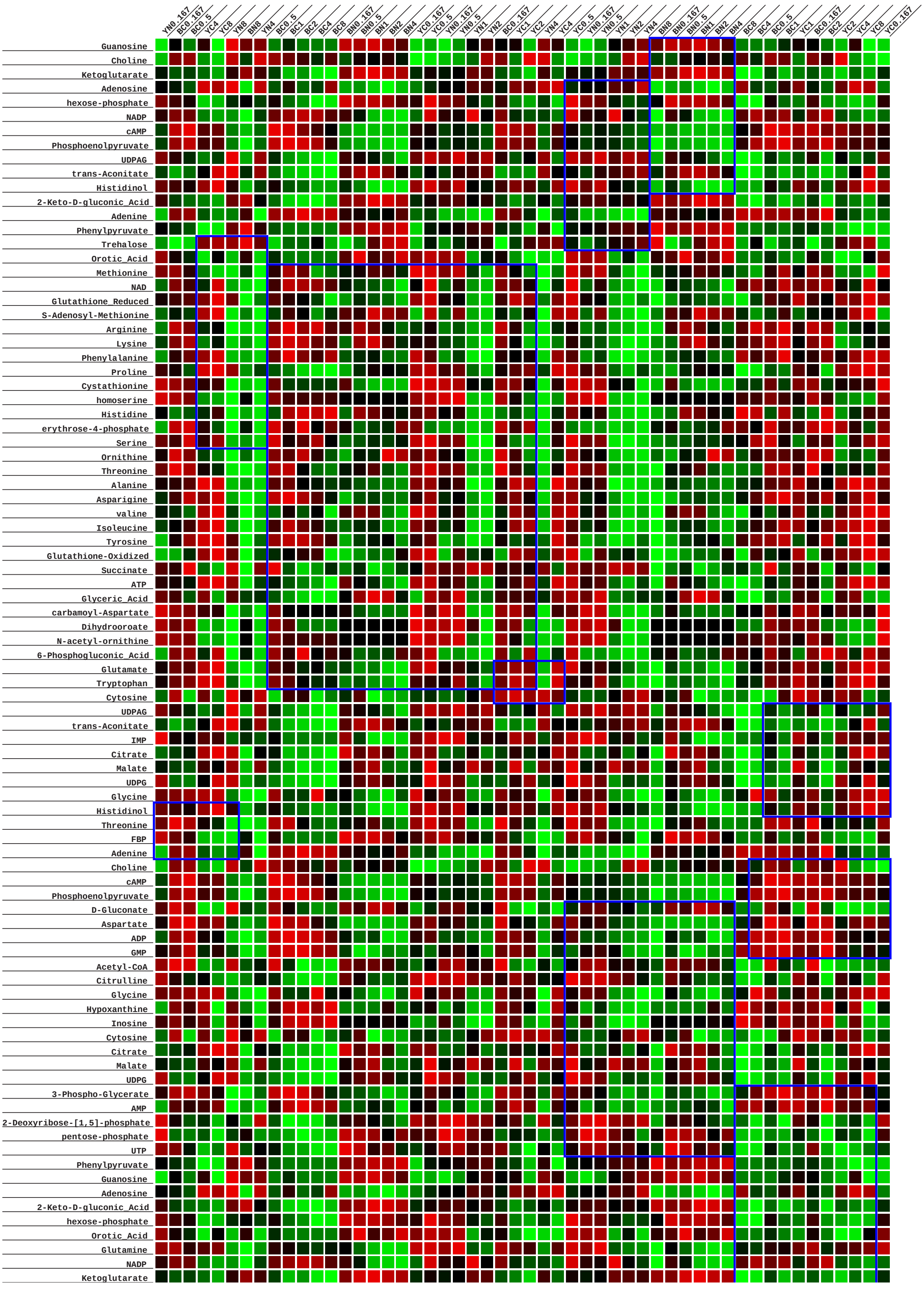

Figure S.1: Biclustering Results for Cheng and Church's Algorithm

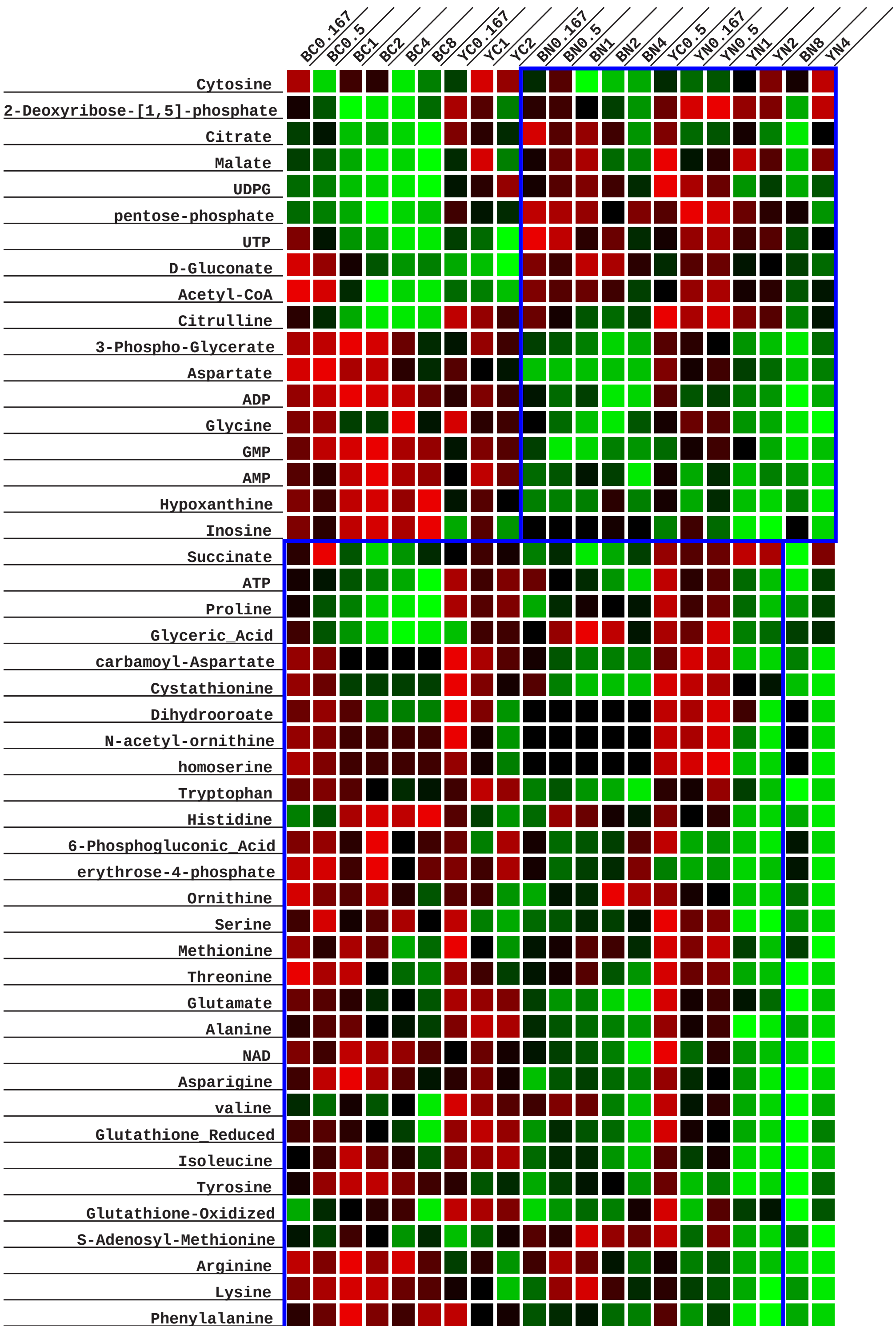

Figure S.2: Biclustering Results for Cheng and Church's Algorithm: Enlarged

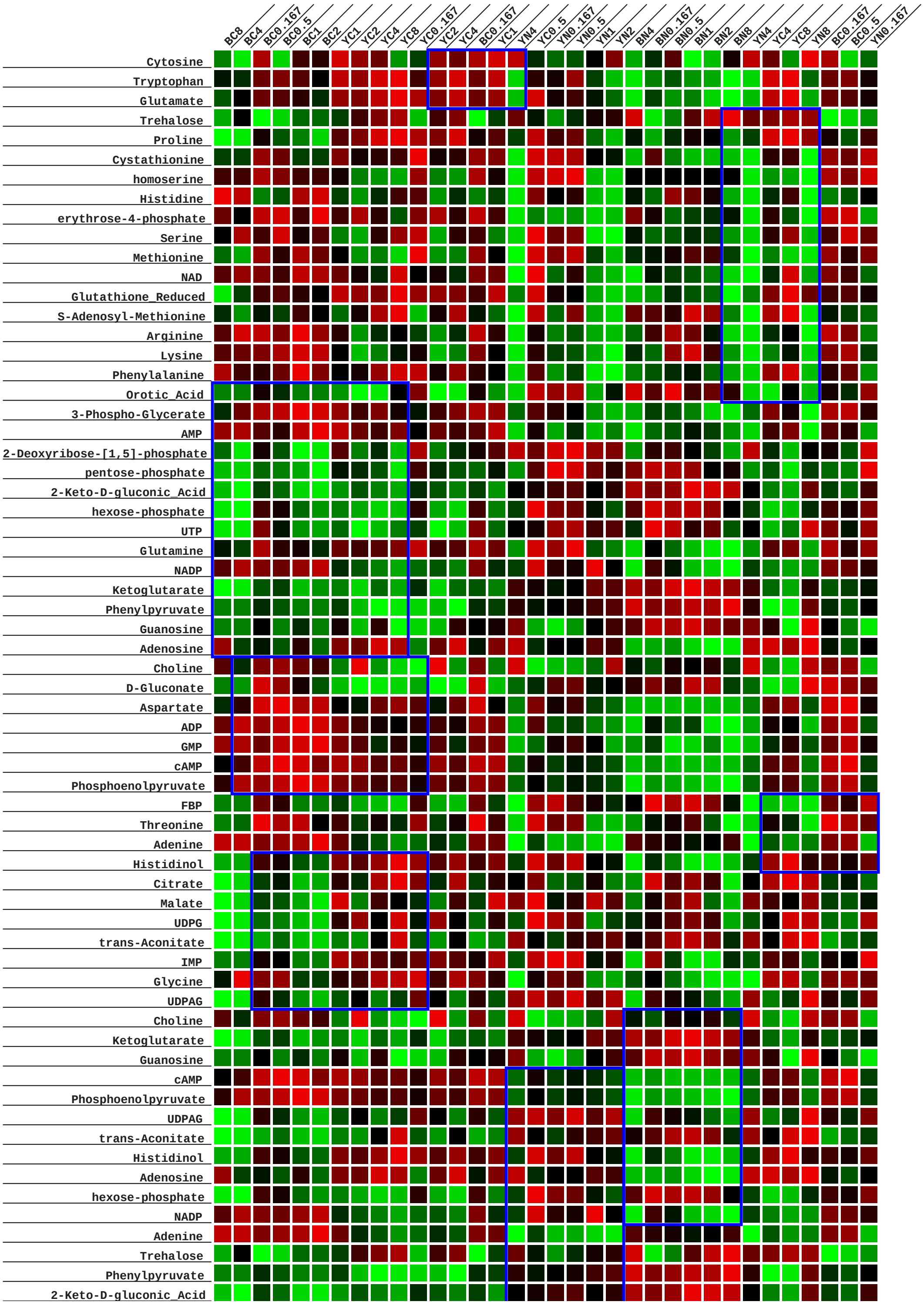

Figure S.3: Biclustering Results for Cheng and Church's Algorithm: Enlarged

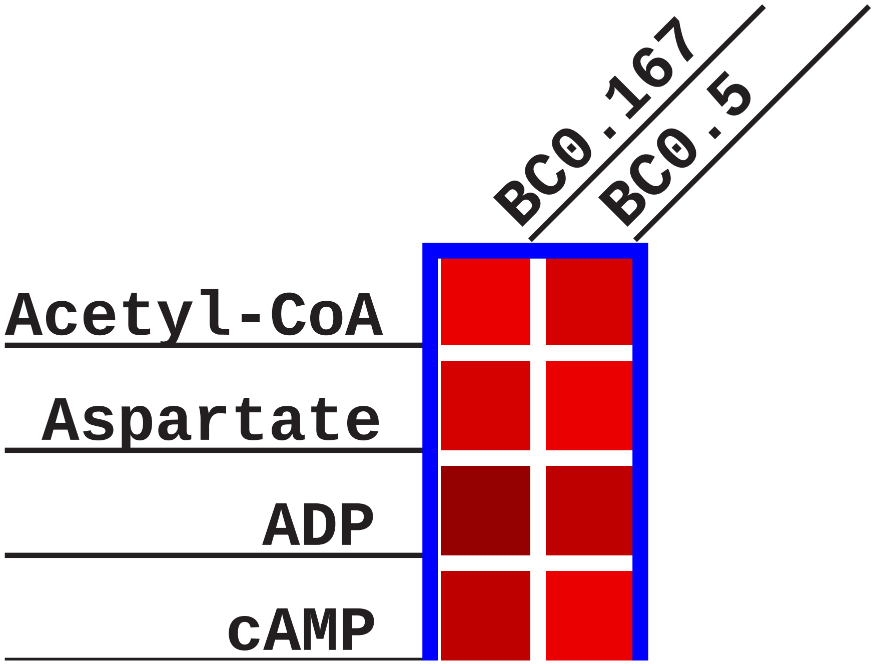

Figure S.4: Biclustering Results for ISA Algorithm

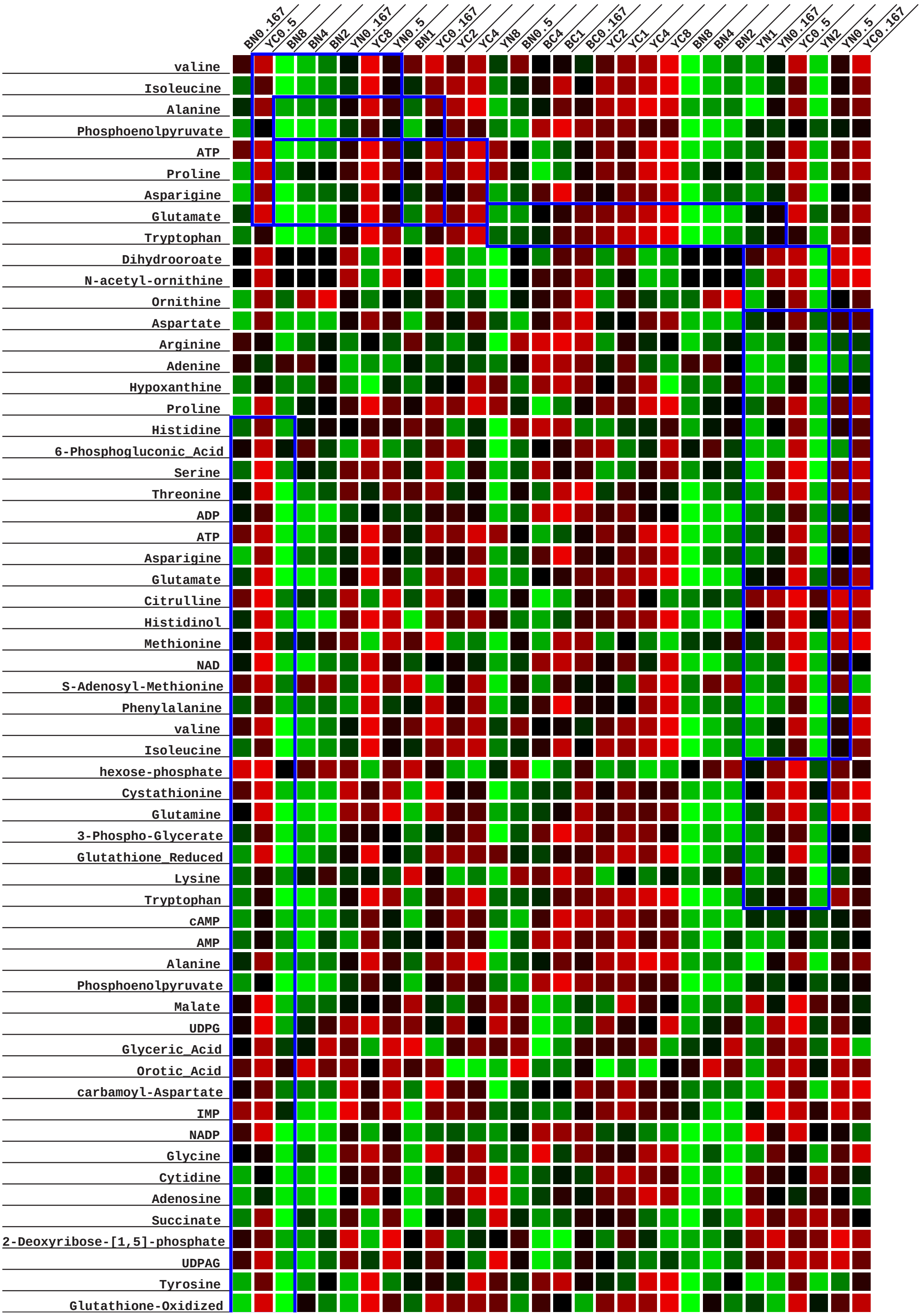

Figure S.5: Biclustering Results for OPSM Algorithm

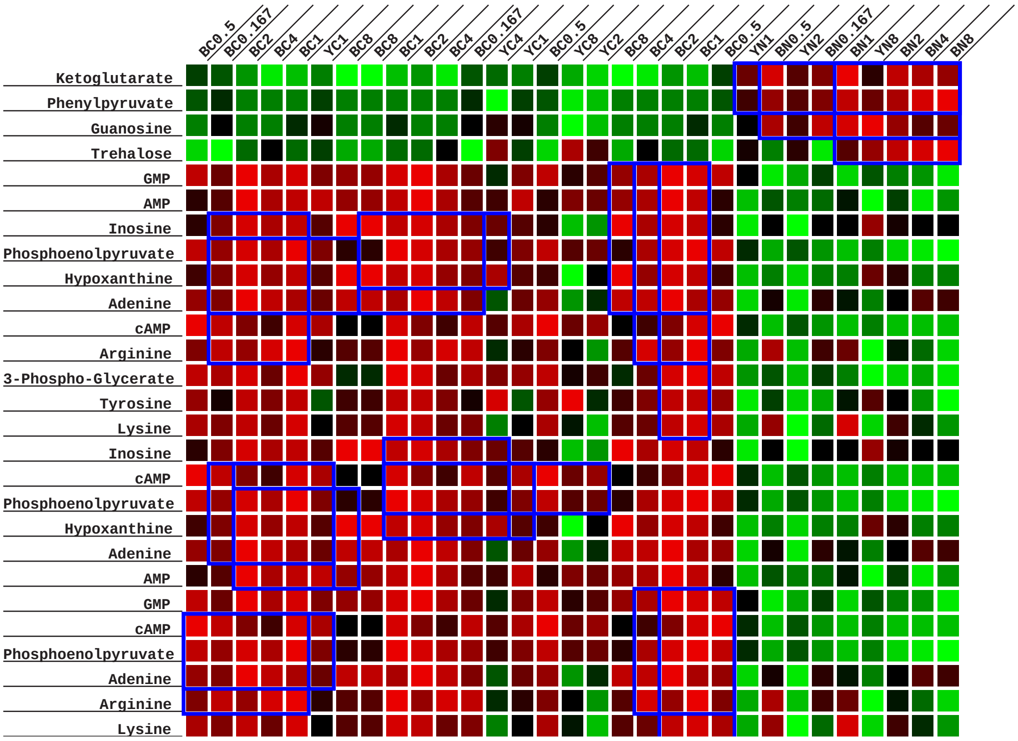

Figure S.6: Biclustering Results for BiMax Algorithm

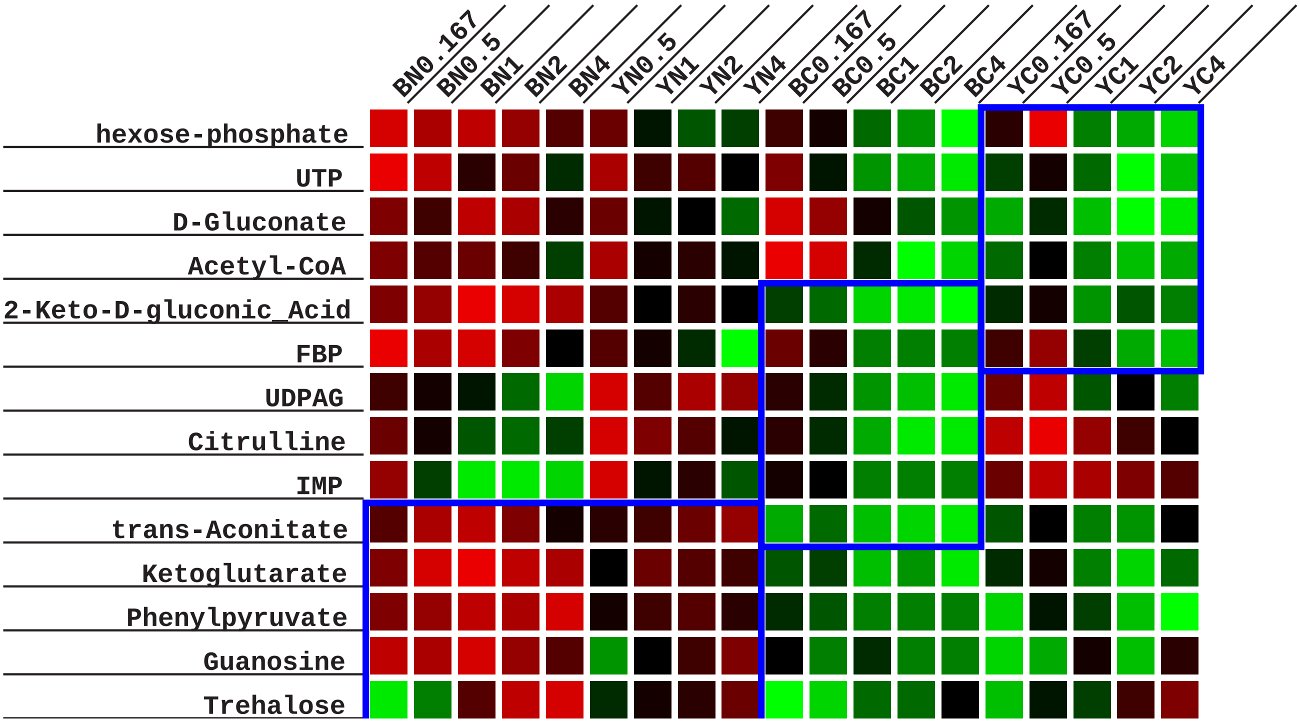

Figure S.7: Biclustering Results for SAMBA Algorithm

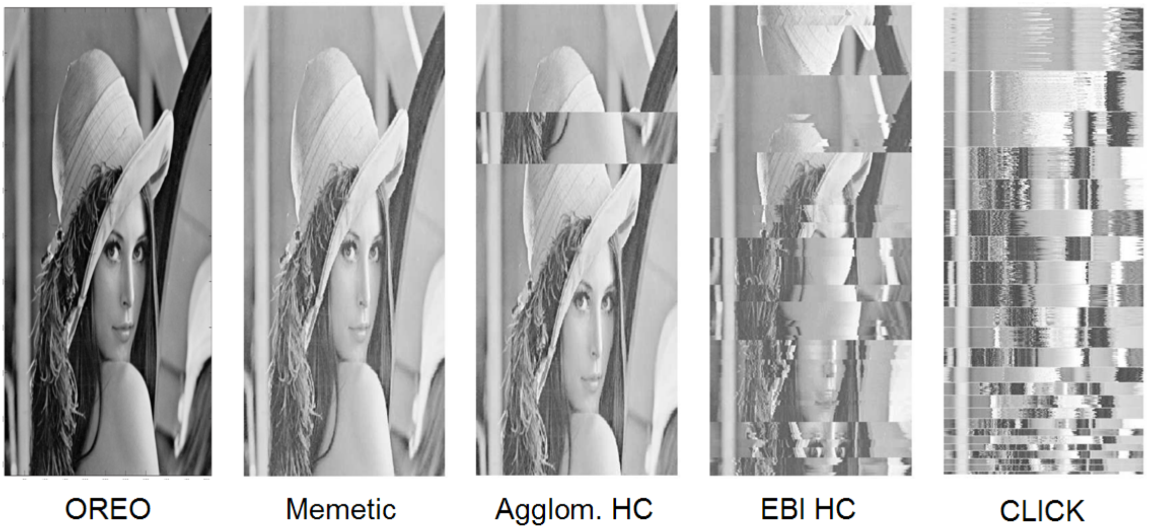

Figure S.8: The clustering results for OREO, a Memetic algorithm, EBI hierarchical clustering, agglomerative hierarchical clustering, and CLICK for the Lenna image replicated row-wise ten times. Note that the only two methods able to recover the correct ordering are OREO and the Memetic algorithm.

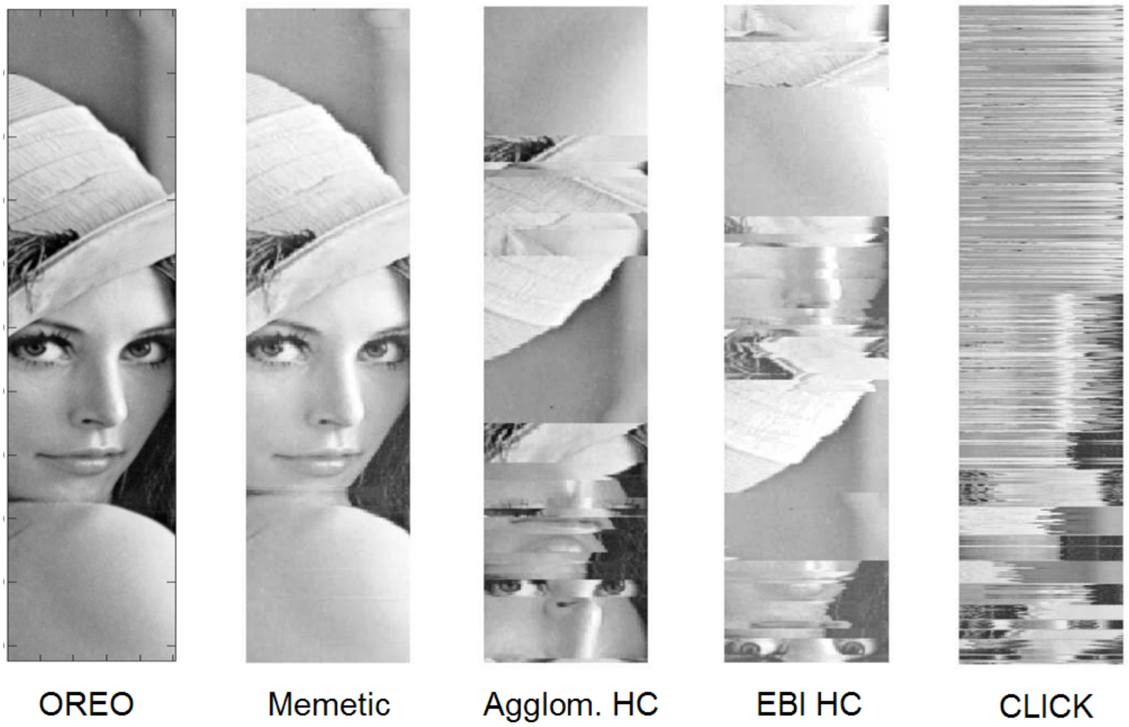

Figure S.9: The clustering results for OREO, a Memetic algorithm, EBI hierarchical clustering, agglomerative hierarchical clustering, and CLICK for the partial Lenna image replicated row-wise ten times. Note that the only two methods able to recover the correct ordering are OREO and the Memetic algorithm.

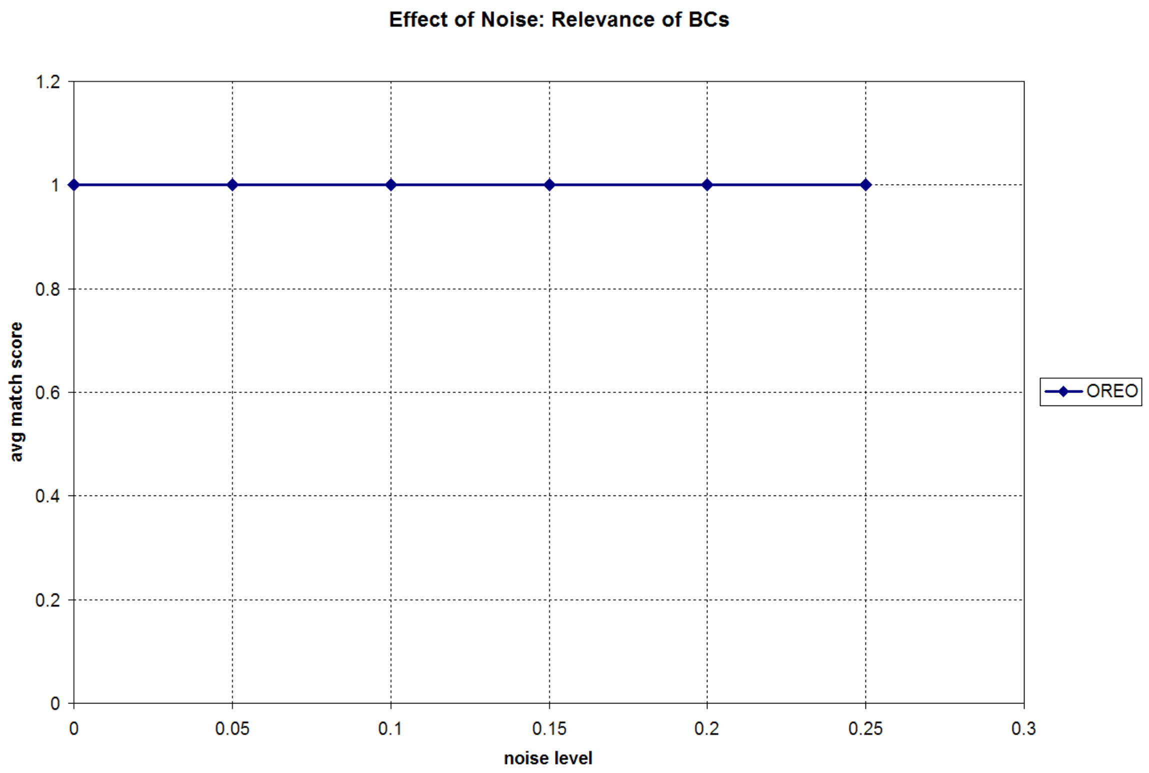

Figure S.10: Average bicluster relevance for OREO for constant, non-overlapping biclusters.

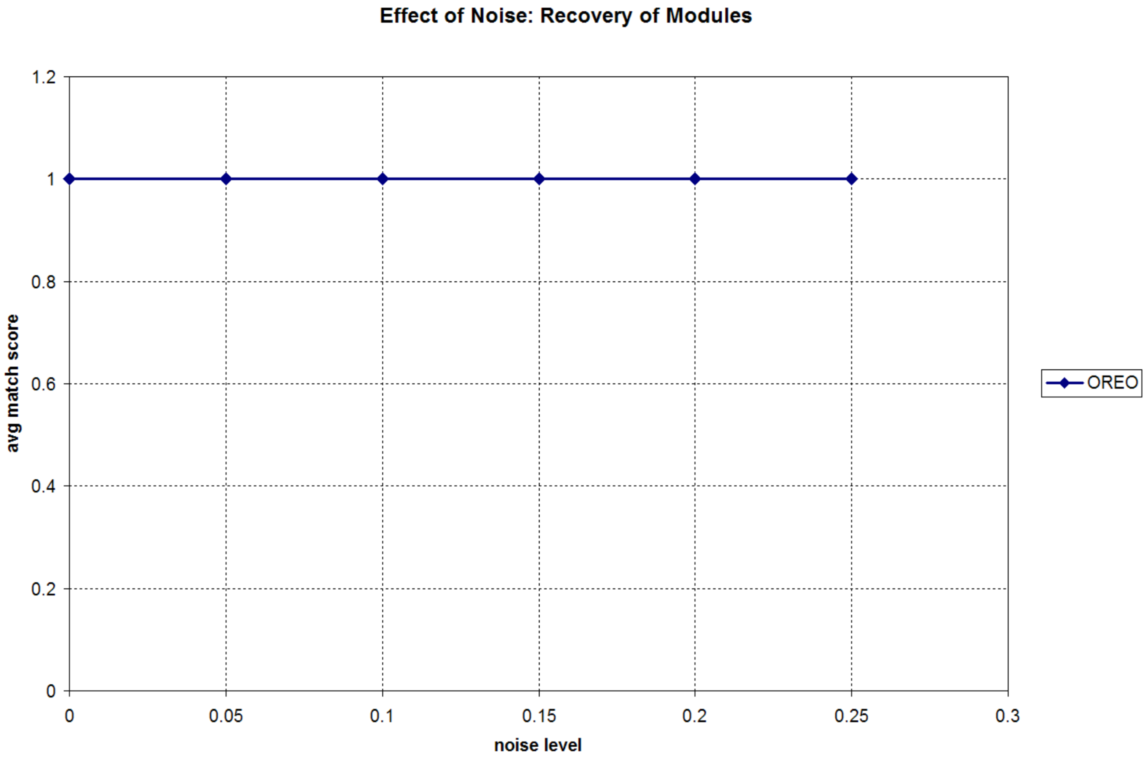

Figure S.11: Average module recovery for OREO for constant, non-overlapping bi-clusters.

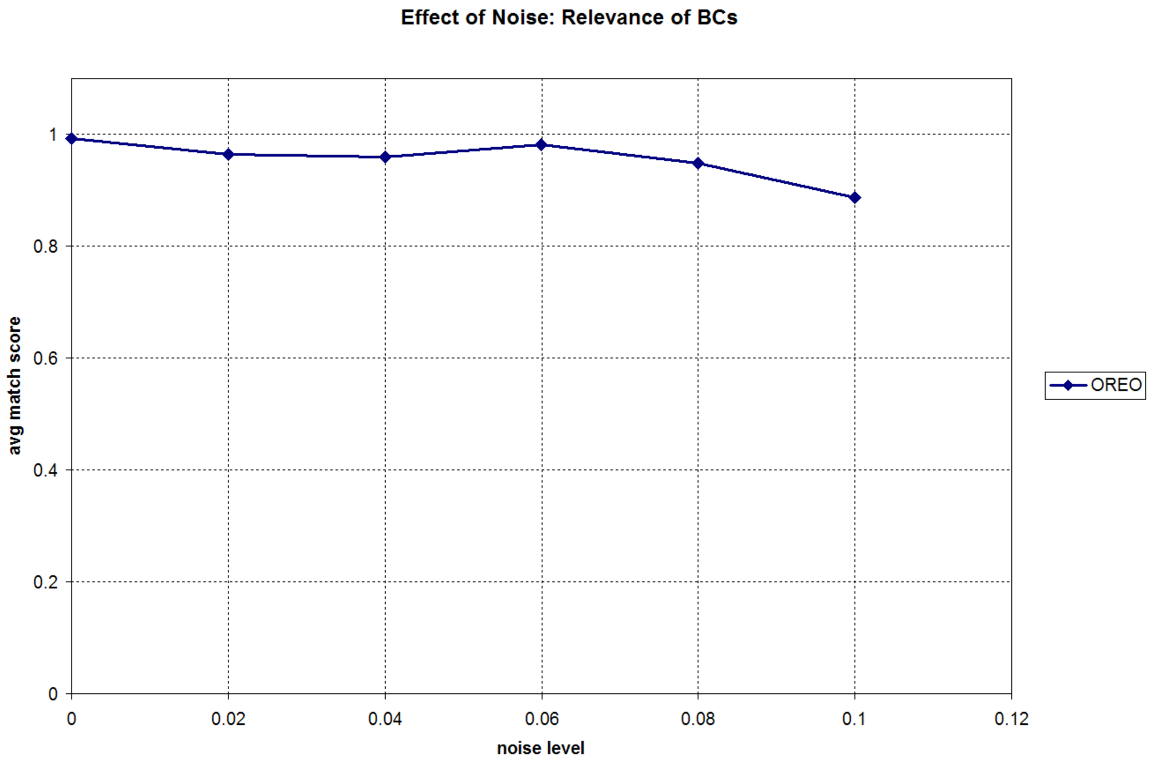

Figure S.12: Average bicluster relevance for OREO for additive, non-overlapping biclusters.

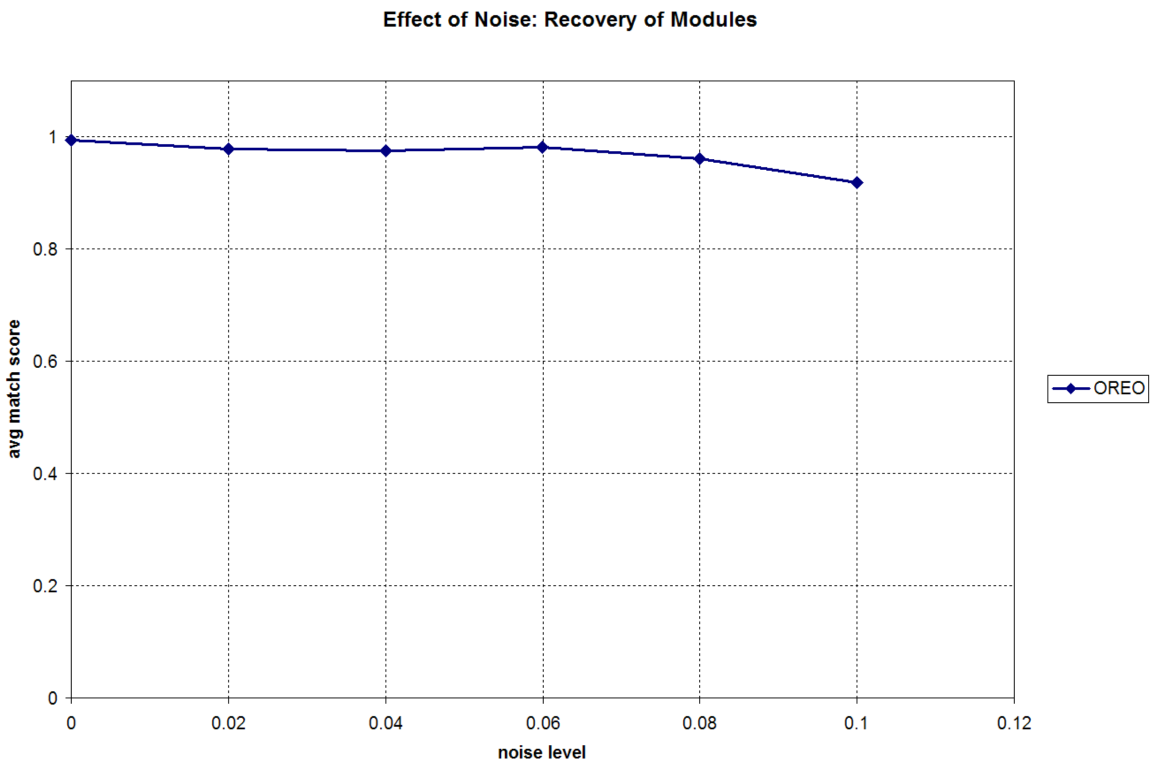

Figure S.13: Average module recovery for OREO for additive, non-overlapping bi-clusters.

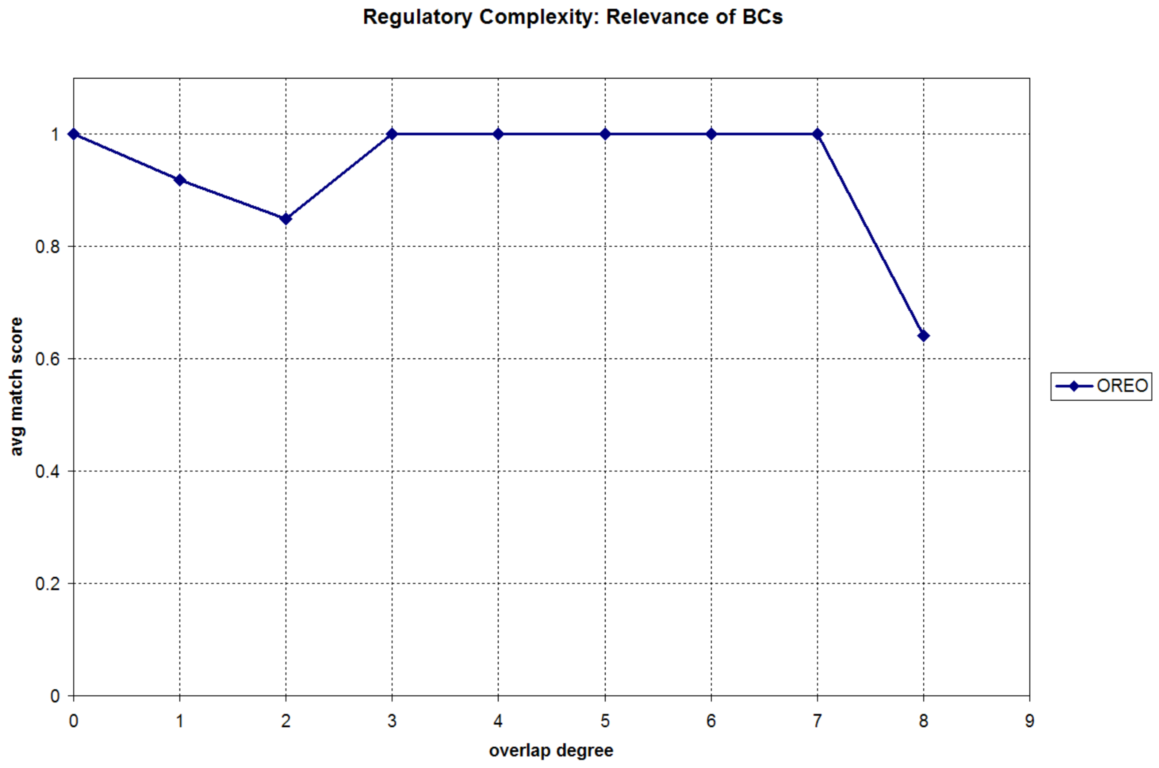

Figure S.14: Average bicluster relevance for OREO for constant, overlapping biclusters.

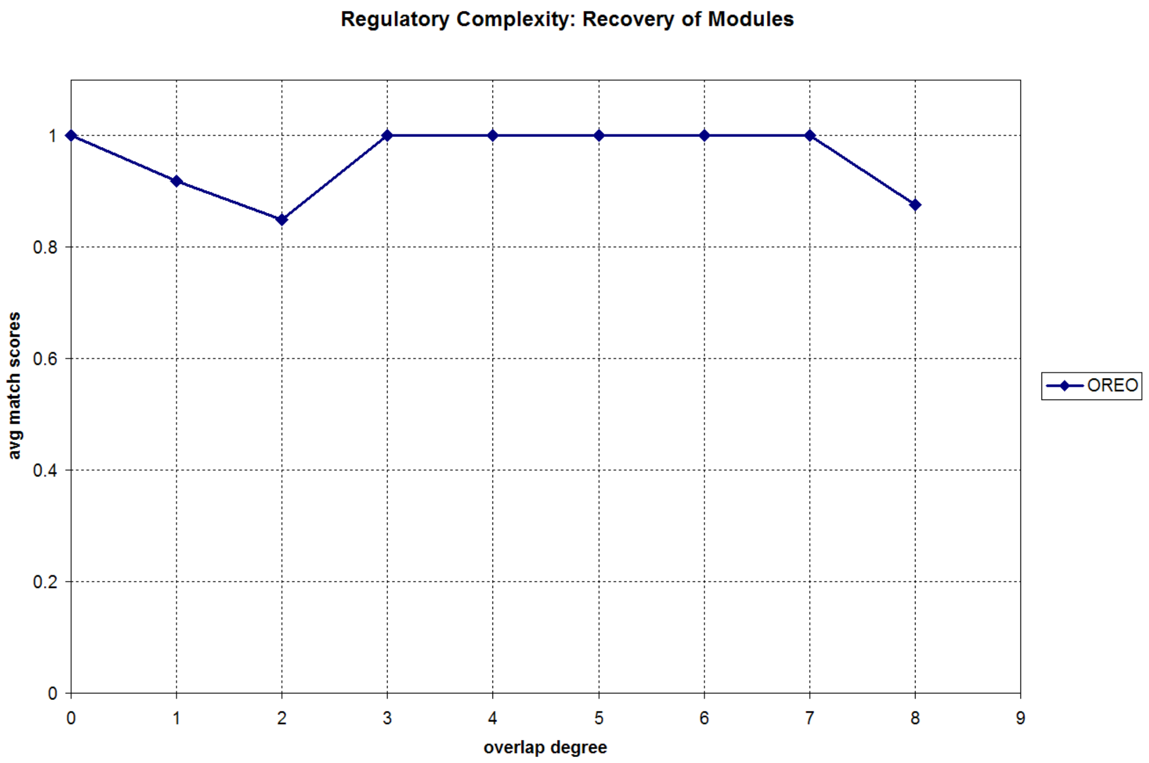

Figure S.15: Average module recovery for OREO for constant, overlapping biclusters.

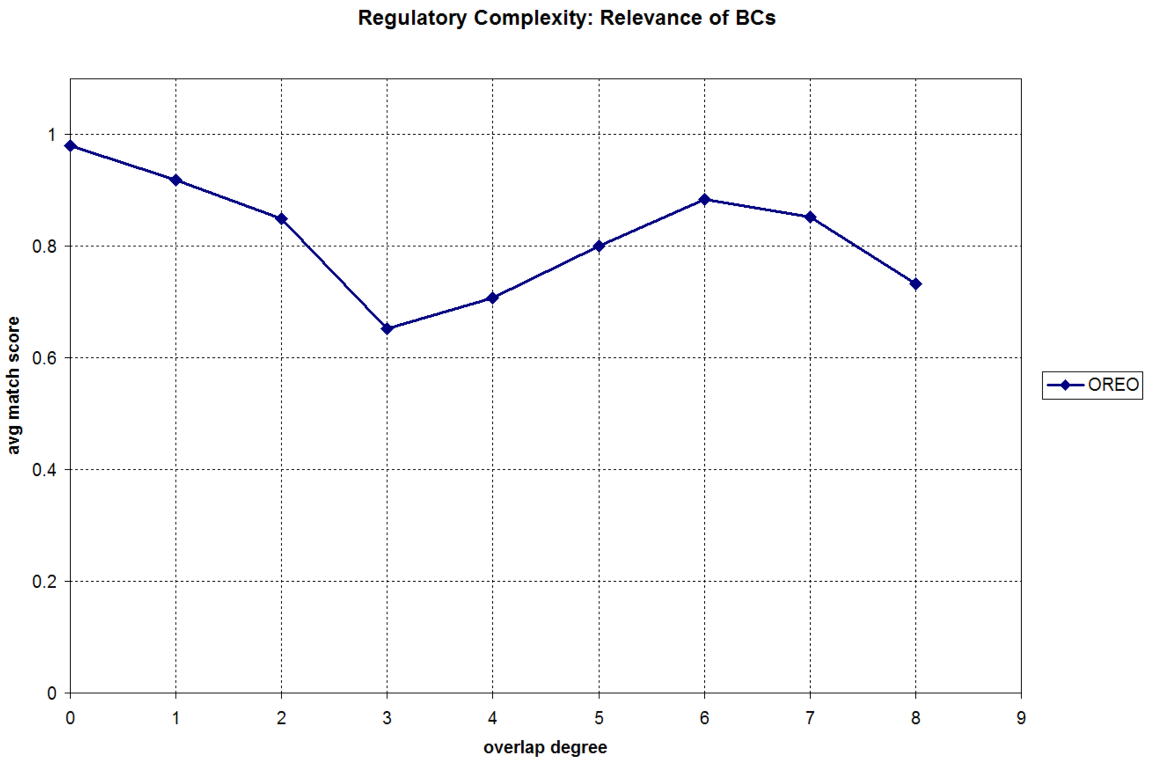

Figure S.16: Average bicluster relevance for OREO for additive, overlapping biclusters.

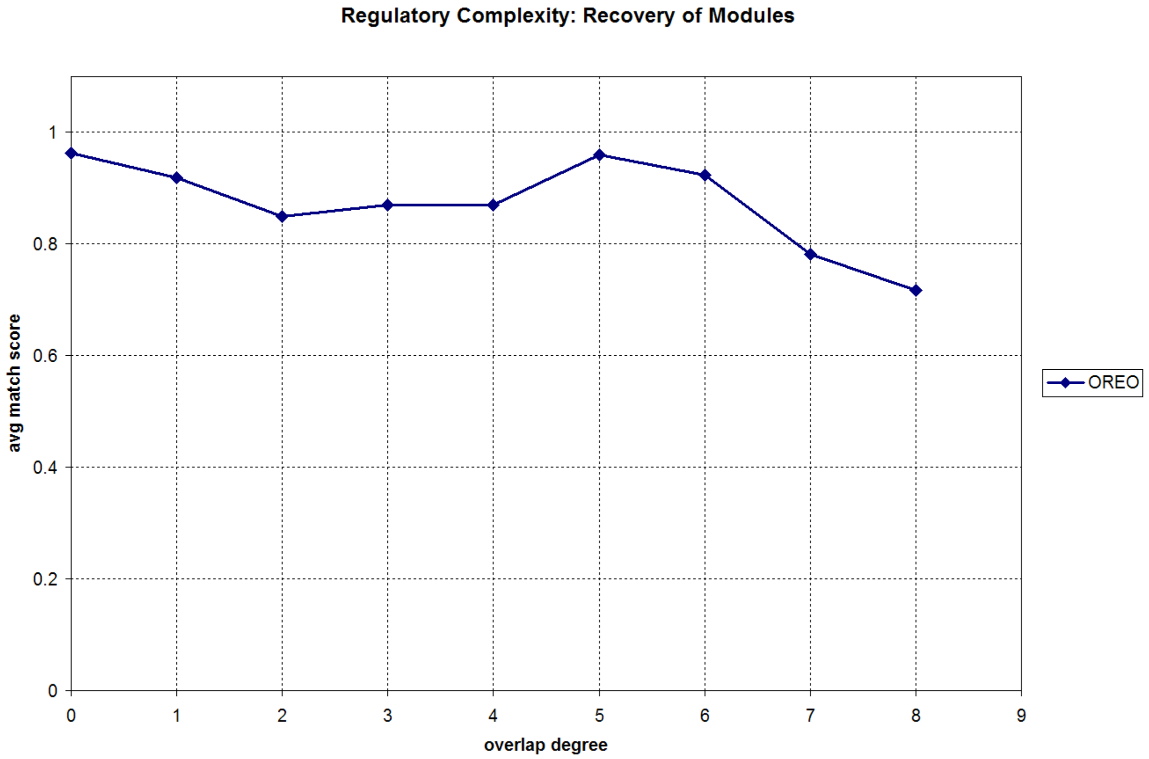

Figure S.17: Average module recovery for OREO for additive, overlapping biclusters.

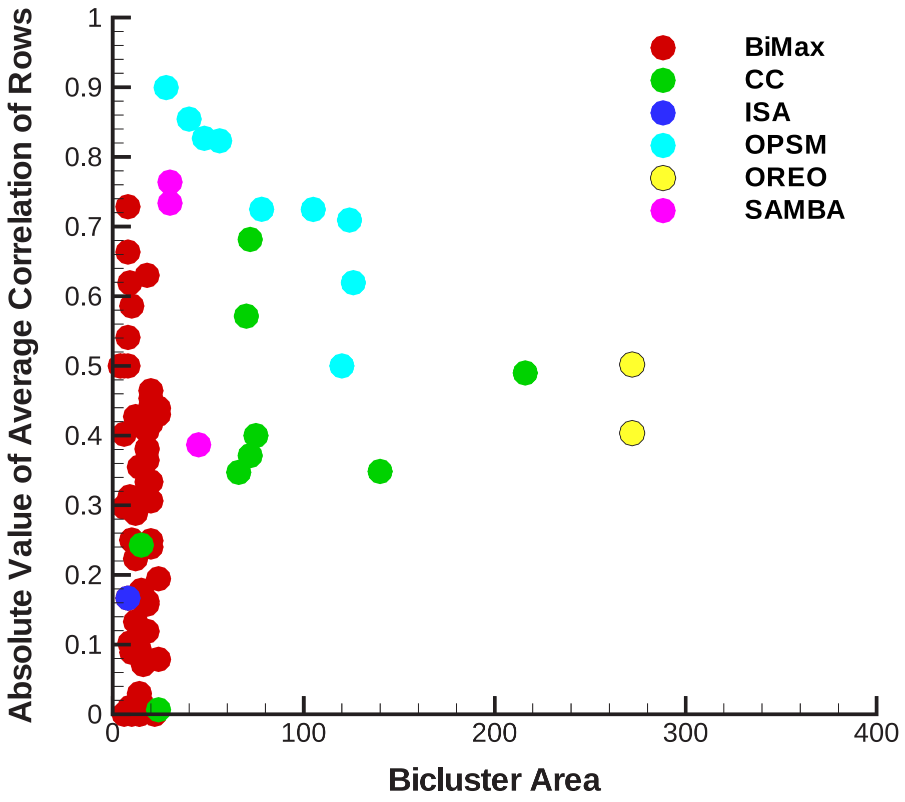

Figure S.18: Absolute value of average correlation values among rows for metabolite concentration data as a function of bicluster area.

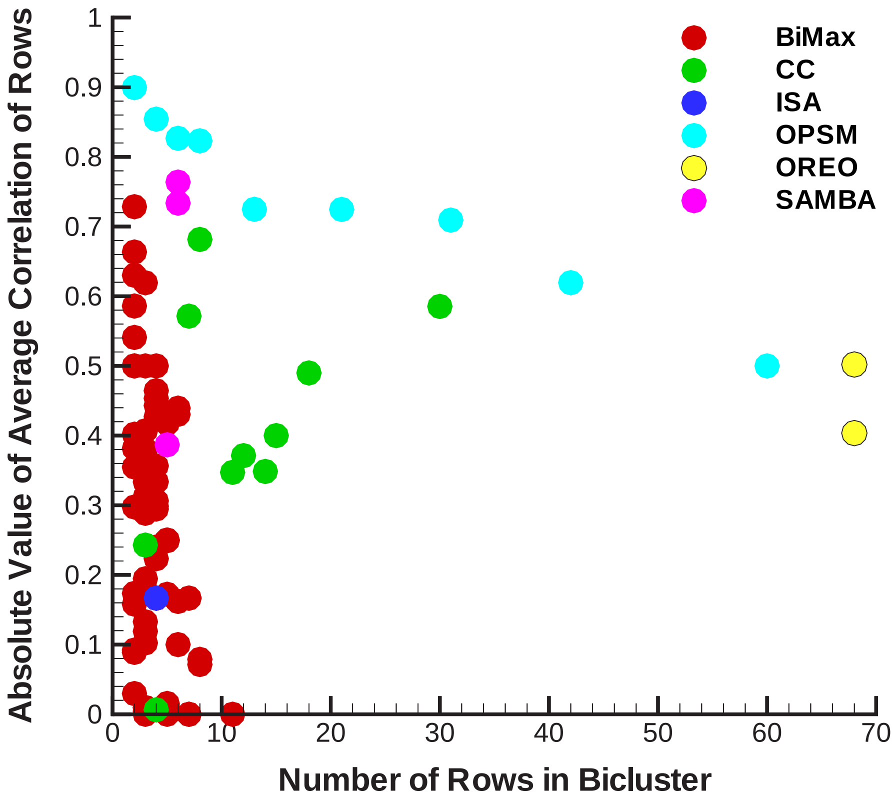

Figure S.19: Absolute value of average correlation values among rows for metabolite concentration data as a function of bicluster row size.

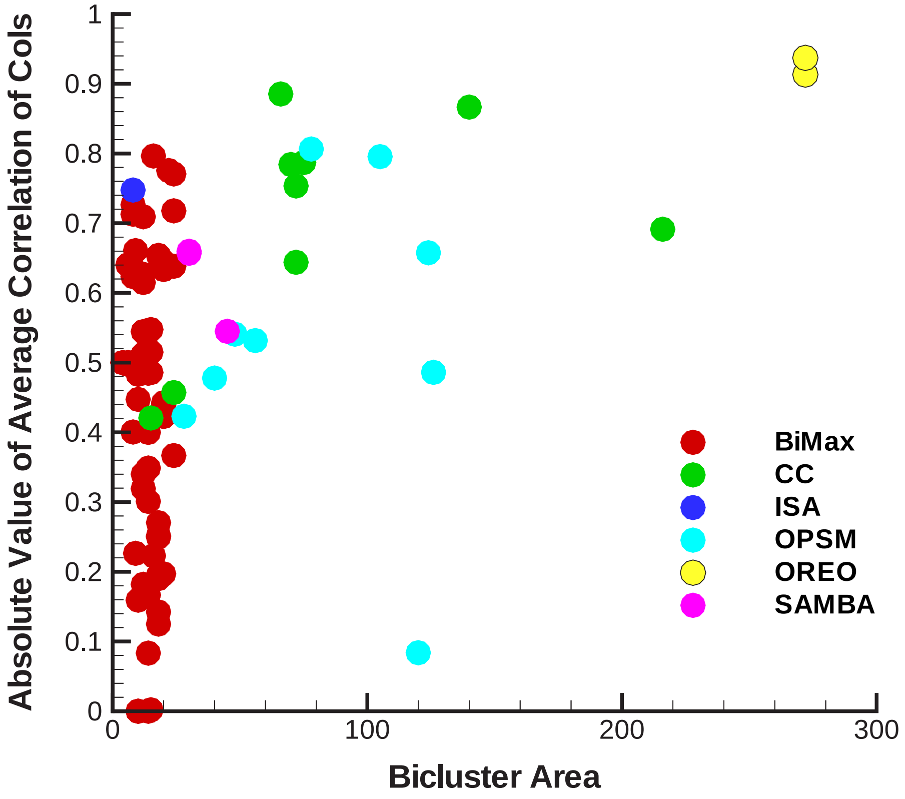

Figure S.20: Absolute value of average correlation values among columns for metabolite concentration data as a function of bicluster area.

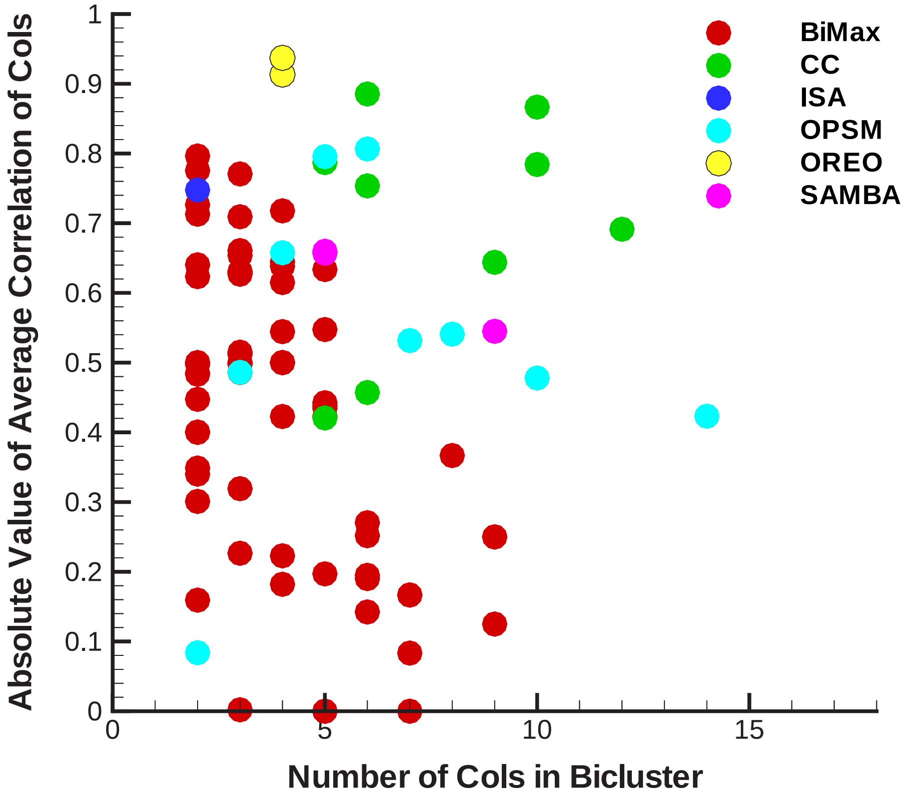

Figure S.21: Absolute value of average correlation values among columns for metabolite concentration data as a function of bicluster column size.

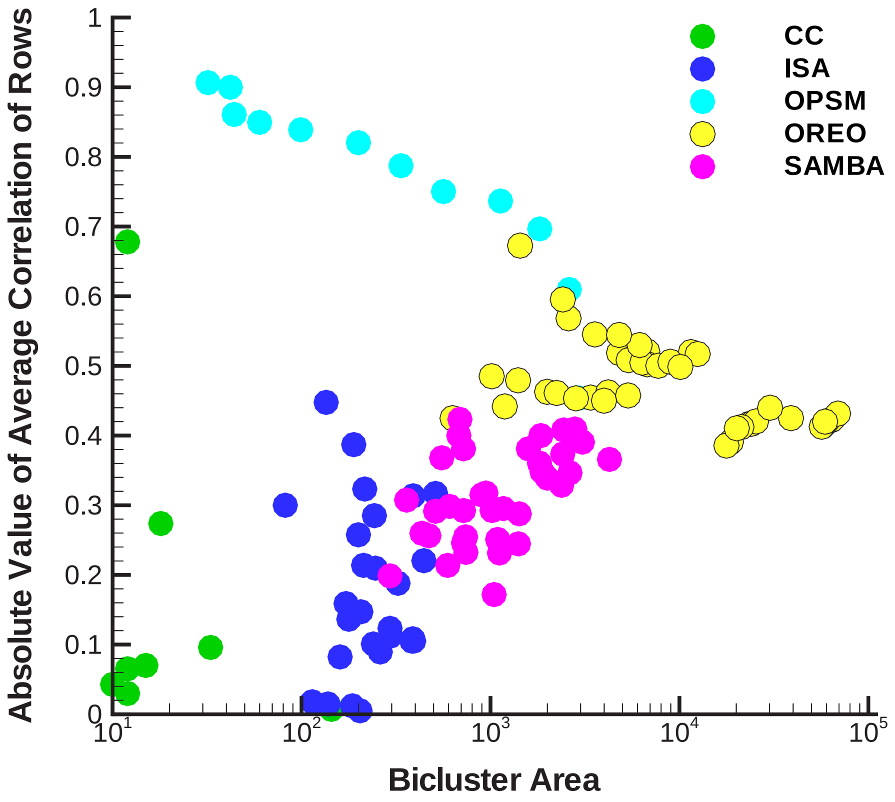

Figure S.22: Absolute value of average correlation values among rows for colon cancer data as a function of bicluster area.

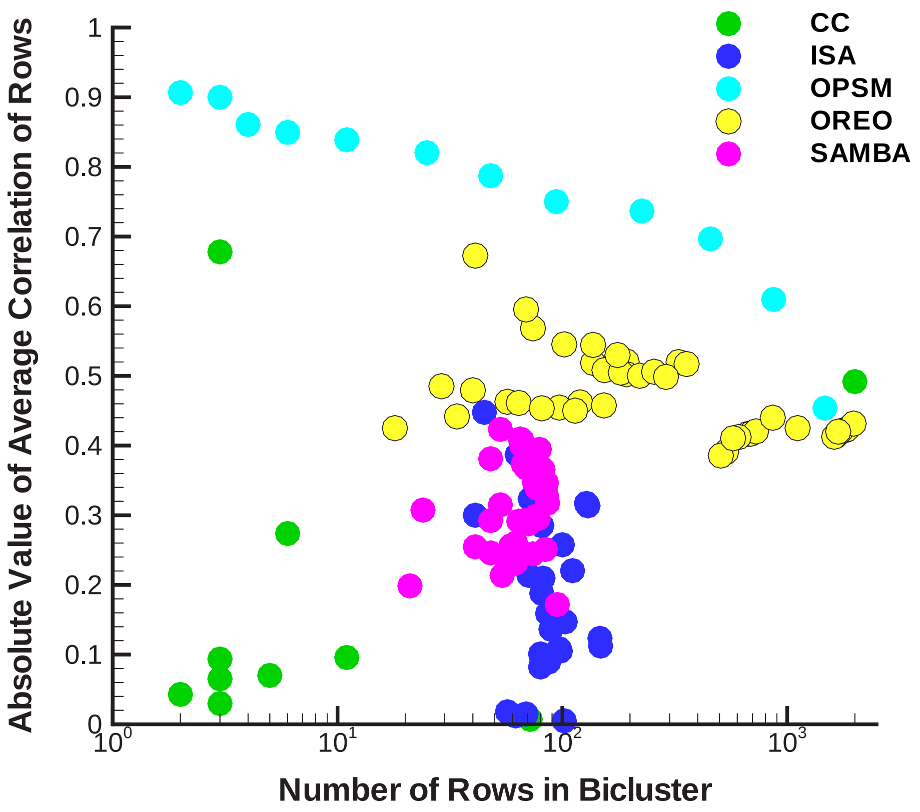

Figure S.23: Absolute value of average correlation values among rows for colon cancer data as a function of bicluster row size.

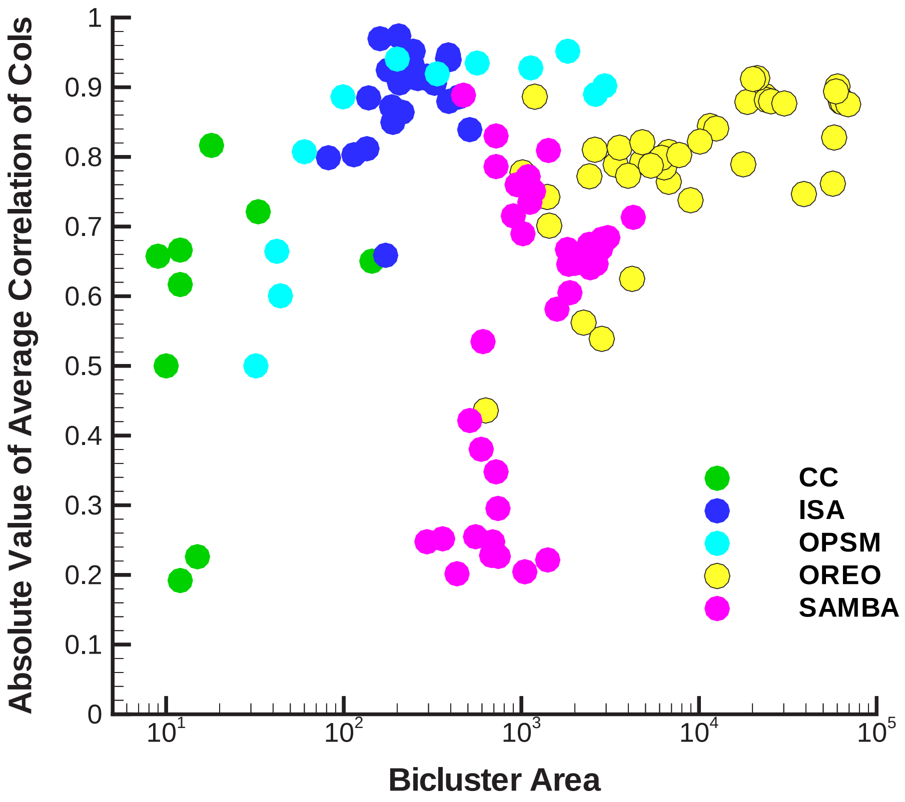

Figure S.24: Absolute value of average correlation values among columns for colon cancer data as a function of bicluster area.

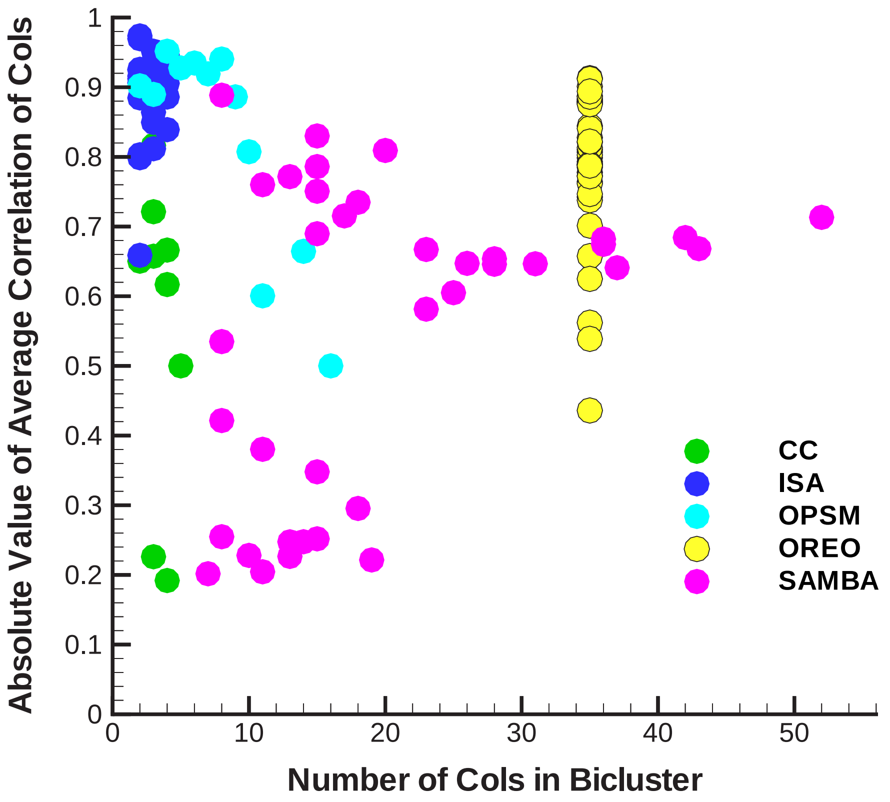

Figure S.25: Absolute value of average correlation values among columns for colon cancer data as a function of bicluster column size.

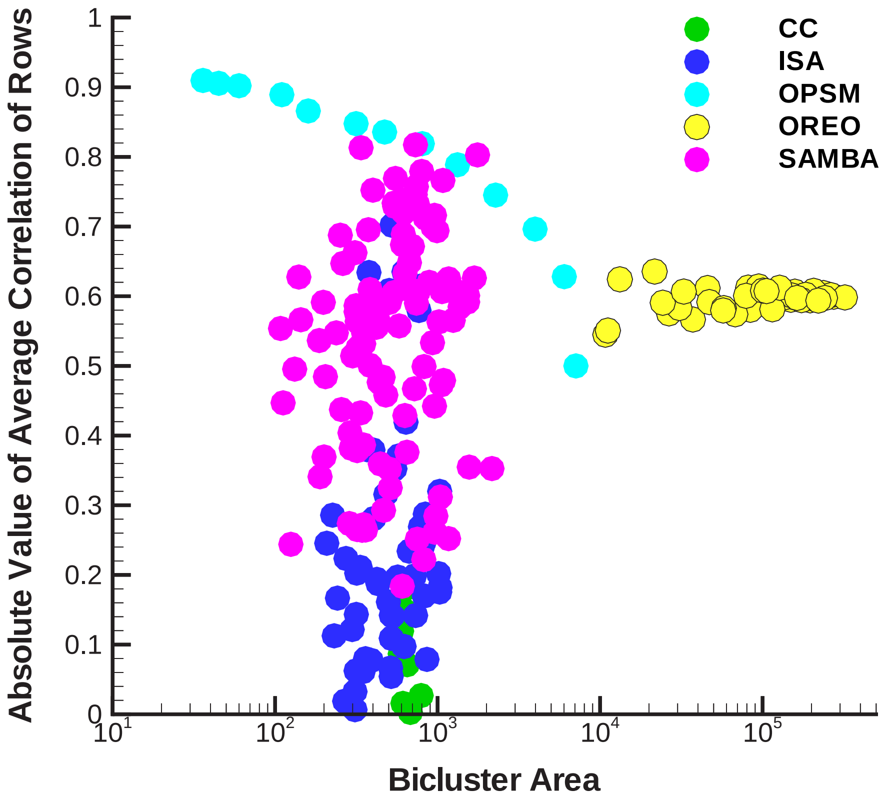

Figure S.26: Absolute value of average correlation values among rows for breast cancer data as a function of bicluster area.

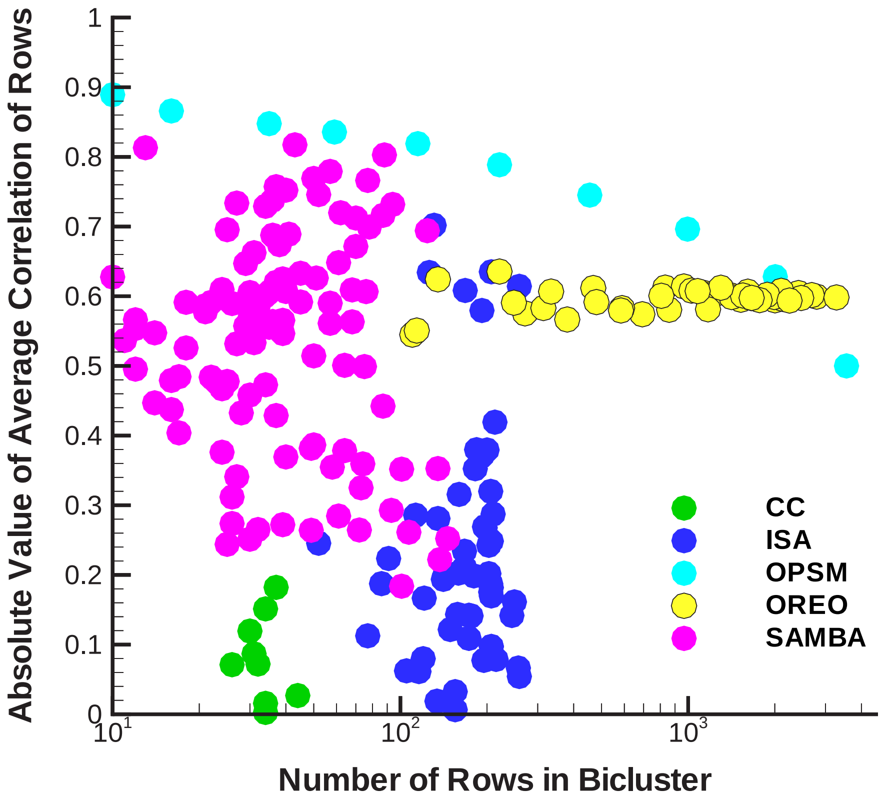

Figure S.27: Absolute value of average correlation values among rows for breast cancer data as a function of bicluster row size.

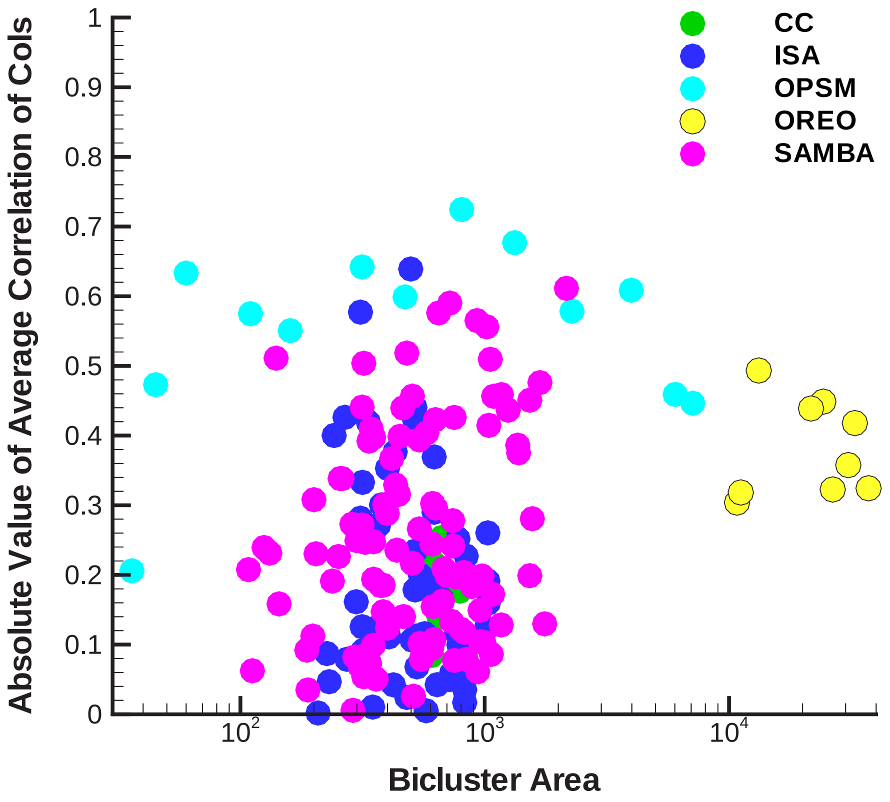

Figure S.28: Absolute value of average correlation values among columns for breast cancer data as a function of bicluster area.

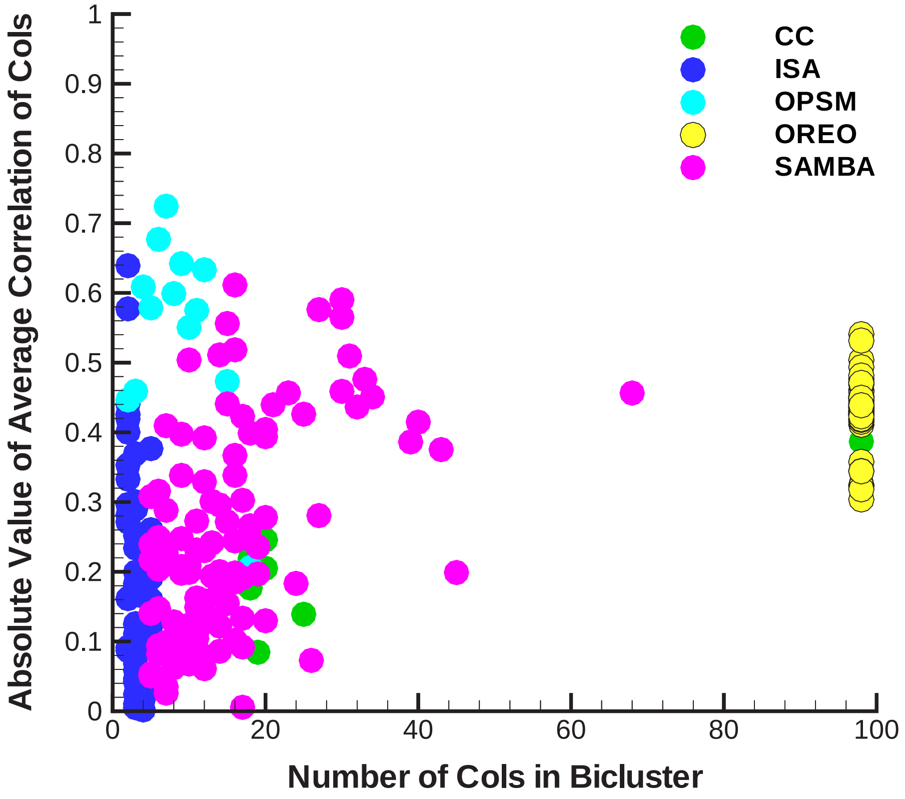

Figure S.29: Absolute value of average correlation values among columns for breast cancer data as a function of bicluster column size.

List of Tables

S.1 Separation of Tumor and Normal Tissues for Alon et al. and OREO for colon cancer data set. The entries in bold indicate a misclassification. 51

Method	Tumor Rich Tissues	Normal Rich Tissues
Alon et al.	T16, T28, T13, T9, T21, T35, T10, T27, T8, T5, T4, T1, T15, T26, T39, T11, T6, T19, T12, T22, T34, T7, **N8**, T3, **N12**, T17, T25, T18, T23, T31, T20, **N34**, T24, T29, T38, T14, T40, T32	N9, **T30**, **T36**, N6, **T33**, N11, N1, N39, N28, N35, N32, N4, N33, N7, **T37**, N5, N27, N3, N2, N40, N36, **T2**, N29, N10
OREO	T2, T37, T25, T17, T14, T15, T18, T4, T1, T11, T19, T23, T16, T5, T10, T34, T24, T26, T12, T9, T21, T6, T20, T29, T31, T32, T8, T13, T22, T38, T28, T35, **N34**, T27, T39, **N8**, **N7**	**T7**, **T3**, N36, **T40**, N40 N33, N27, N35, **T36**, N28, N29, **T30**, N32, N39, **T33**, N1, N6, N2, N11, N5, N4, N3, N10, N9, N12

Table S.1: Separation of Tumor and Normal Tissues for Alon et al. and OREO for colon cancer data set. The entries in bold indicate a misclassification.
